# Supplementary material for: Brassinosteroids control cell proliferation in the lateral root cap of the Arabidopsis root
Source: EMBO Rep. 2026 Apr 10;27(9):2183–200. doi: 10.1038/s44319-026-00737-0 (PMC13172465; doi:10.1038/s44319-026-00737-0)
Supplement: Supplementary file 2 — Source data Fig. 1 [file 44319_2026_737_MOESM2_ESM.zip › Figure 1/1B/README.rtf]

Confocal images of the root tip of J3411. Overlapping tiles are labeled sequentially name_1, name_2.
