# Supplementary figures and images for: Brassinosteroids control cell proliferation in the lateral root cap of the Arabidopsis root
Source: EMBO Rep. 2026 Apr 10;27(9):2183–200. doi: 10.1038/s44319-026-00737-0 (PMC13172465; doi:10.1038/s44319-026-00737-0)

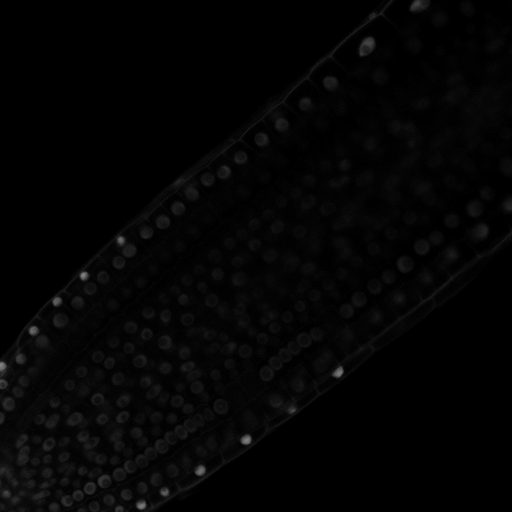

Supplement: Supplementary file 2 — Source data Fig. 1 [file 44319_2026_737_MOESM2_ESM.zip › Figure 1/1D/Timer-NLS_2.tif]

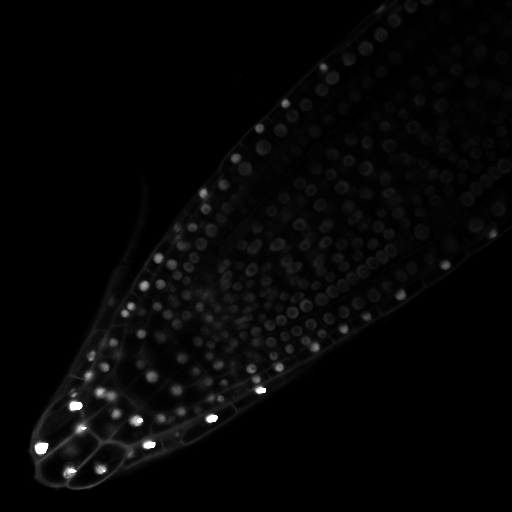

Supplement: Supplementary file 2 — Source data Fig. 1 [file 44319_2026_737_MOESM2_ESM.zip › Figure 1/1D/Timer-NLS_1.tif]

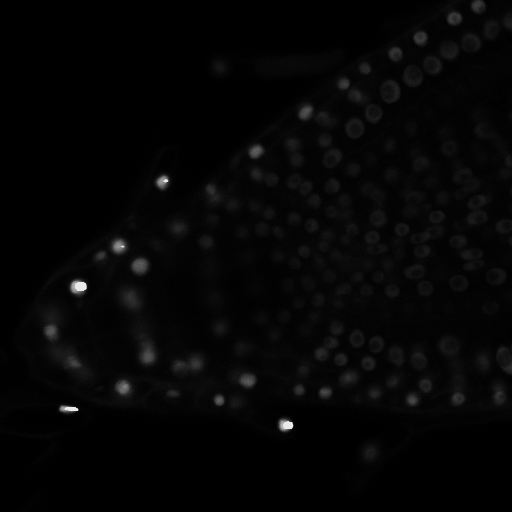

Supplement: Supplementary file 3 — Source data Fig. 2 [file 44319_2026_737_MOESM3_ESM.zip › Figure 2/2G/+PPZ.tif]

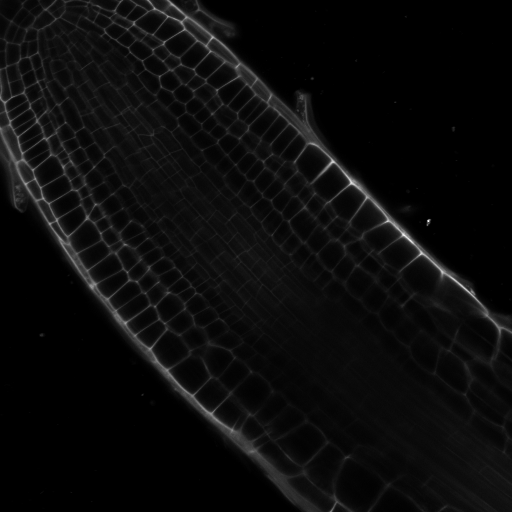

Supplement: Supplementary file 3 — Source data Fig. 2 [file 44319_2026_737_MOESM3_ESM.zip › Figure 2/2A/bri3_2.tif]

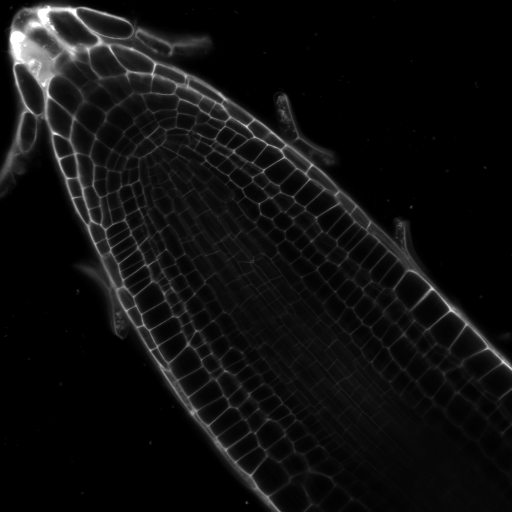

Supplement: Supplementary file 3 — Source data Fig. 2 [file 44319_2026_737_MOESM3_ESM.zip › Figure 2/2A/bri3_1.tif]

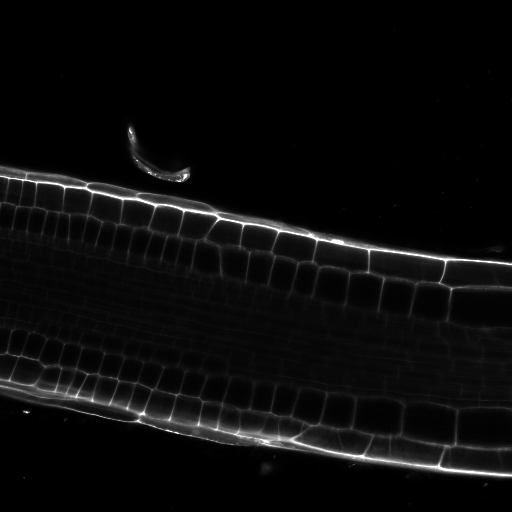

Supplement: Supplementary file 3 — Source data Fig. 2 [file 44319_2026_737_MOESM3_ESM.zip › Figure 2/2A/WT_2.tif]

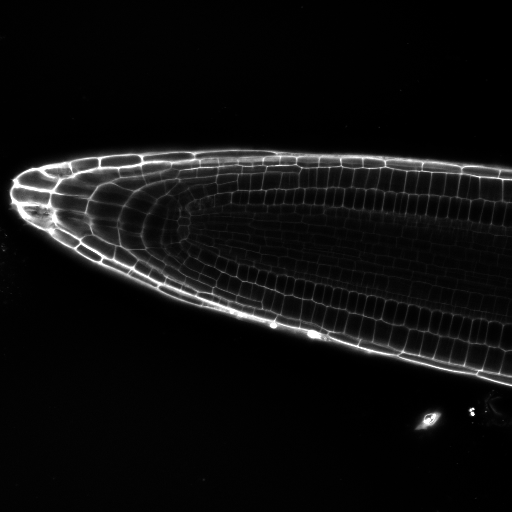

Supplement: Supplementary file 3 — Source data Fig. 2 [file 44319_2026_737_MOESM3_ESM.zip › Figure 2/2A/WT_1.tif]

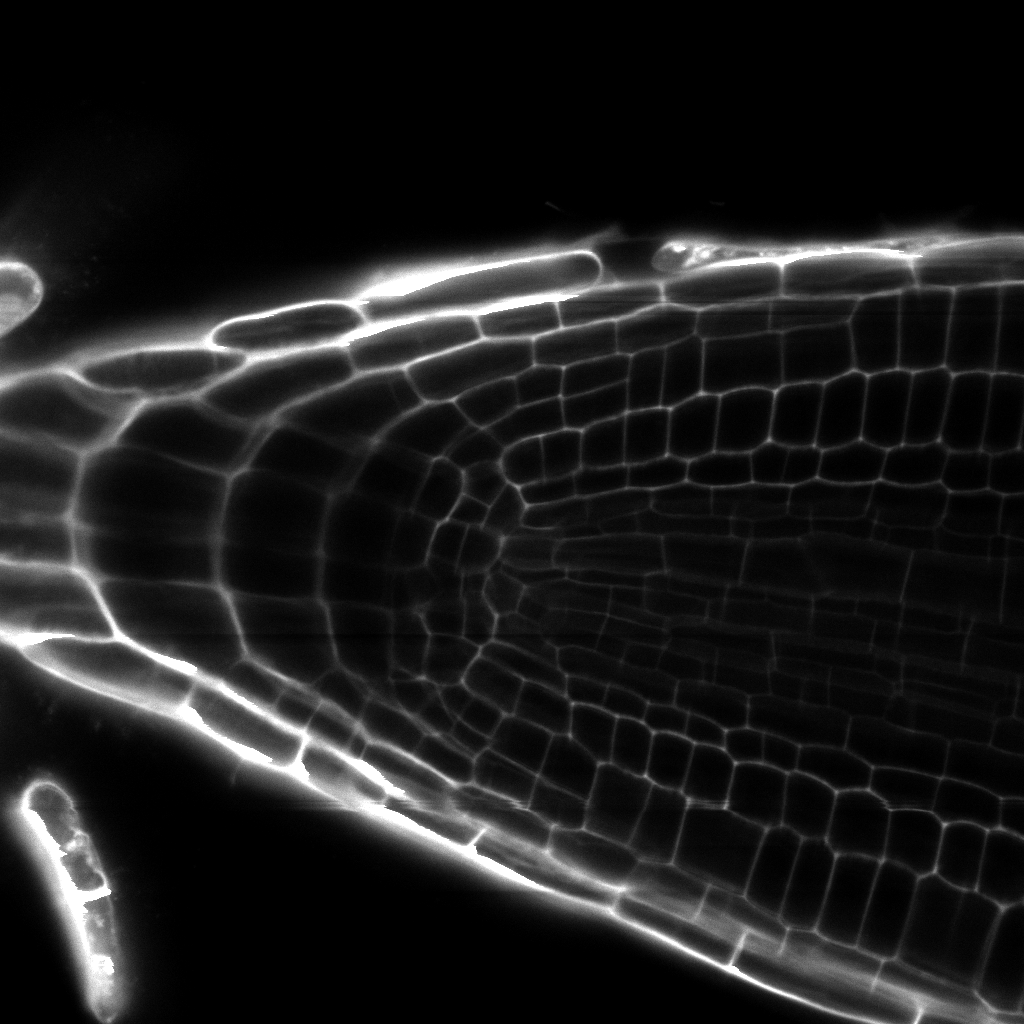

Supplement: Supplementary file 4 — Source data Fig. 3 [file 44319_2026_737_MOESM4_ESM.zip › Figure 3/3E/WT.tif]

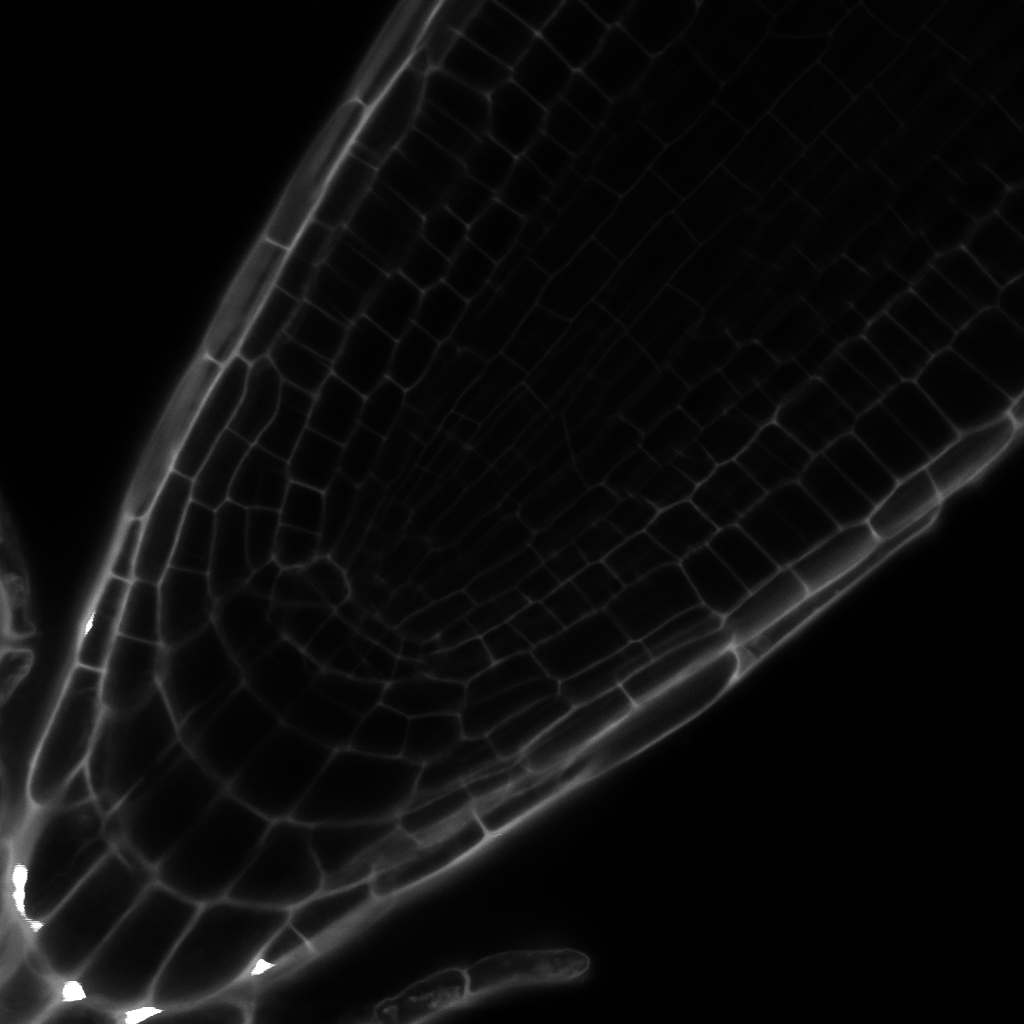

Supplement: Supplementary file 4 — Source data Fig. 3 [file 44319_2026_737_MOESM4_ESM.zip › Figure 3/3E/bzr1-2.tif]

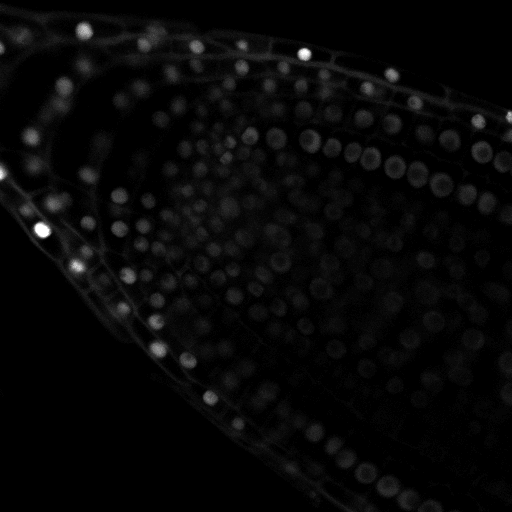

Supplement: Supplementary file 4 — Source data Fig. 3 [file 44319_2026_737_MOESM4_ESM.zip › Figure 3/3G/bzr1-2.tif]

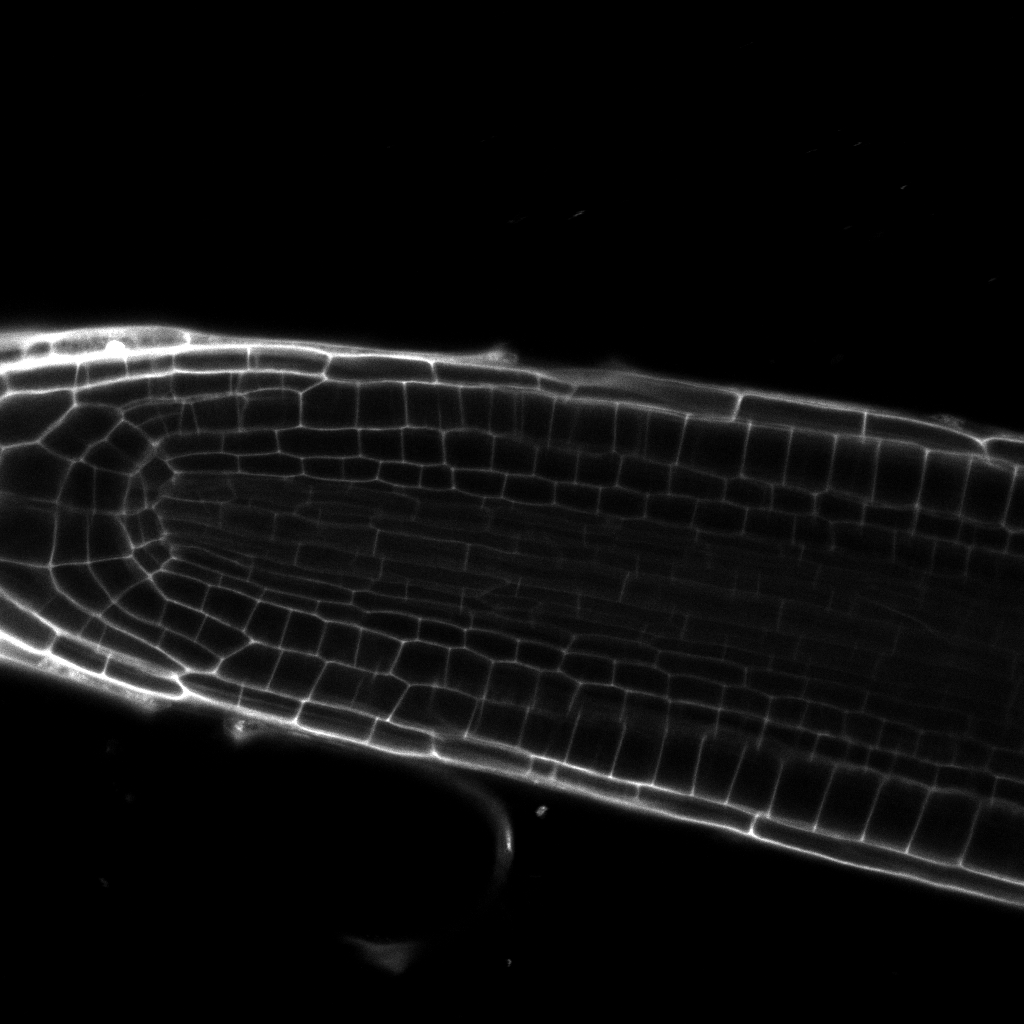

Supplement: Supplementary file 5 — Source data Fig. 4 [file 44319_2026_737_MOESM5_ESM.zip › Figure 4/4E/cycd3;3_BL2.tif]

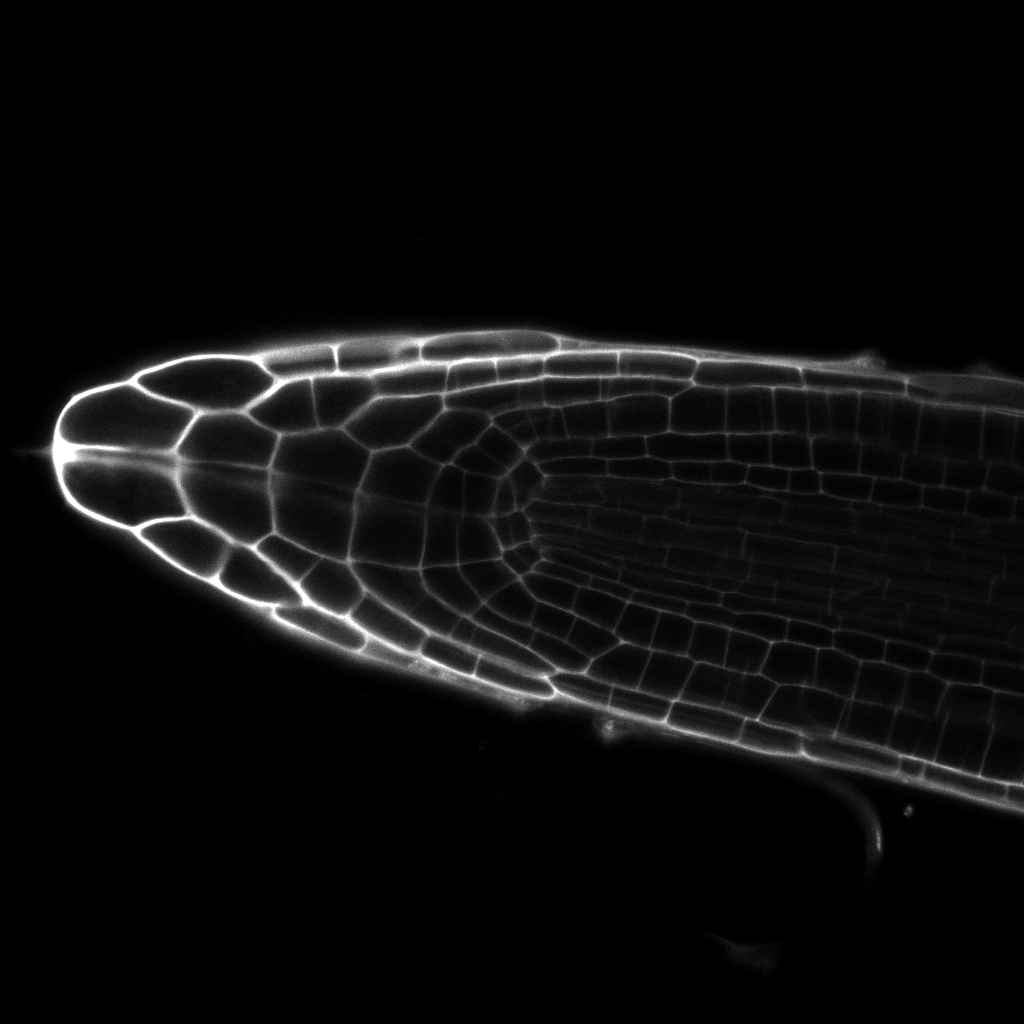

Supplement: Supplementary file 5 — Source data Fig. 4 [file 44319_2026_737_MOESM5_ESM.zip › Figure 4/4E/cycd3;3_BL1.tif]

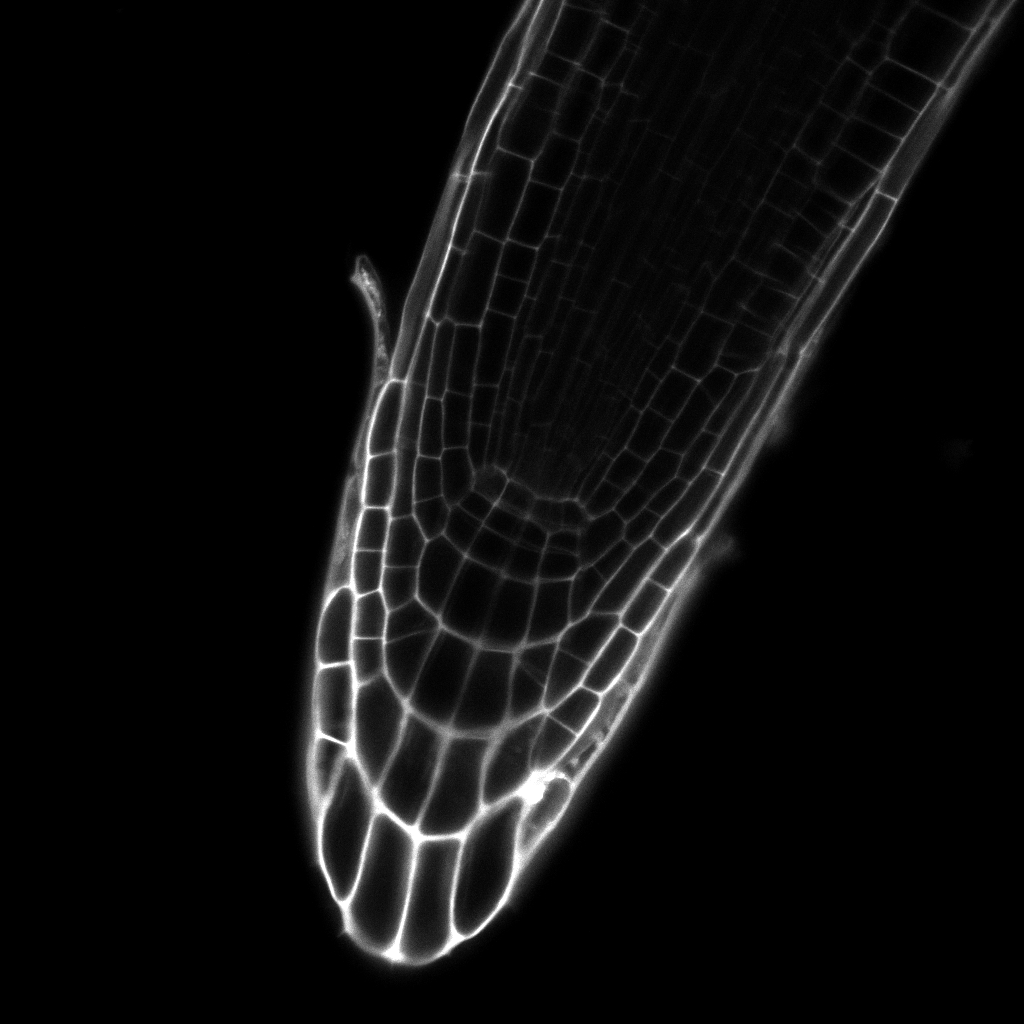

Supplement: Supplementary file 5 — Source data Fig. 4 [file 44319_2026_737_MOESM5_ESM.zip › Figure 4/4E/WT_BL_2.tif]

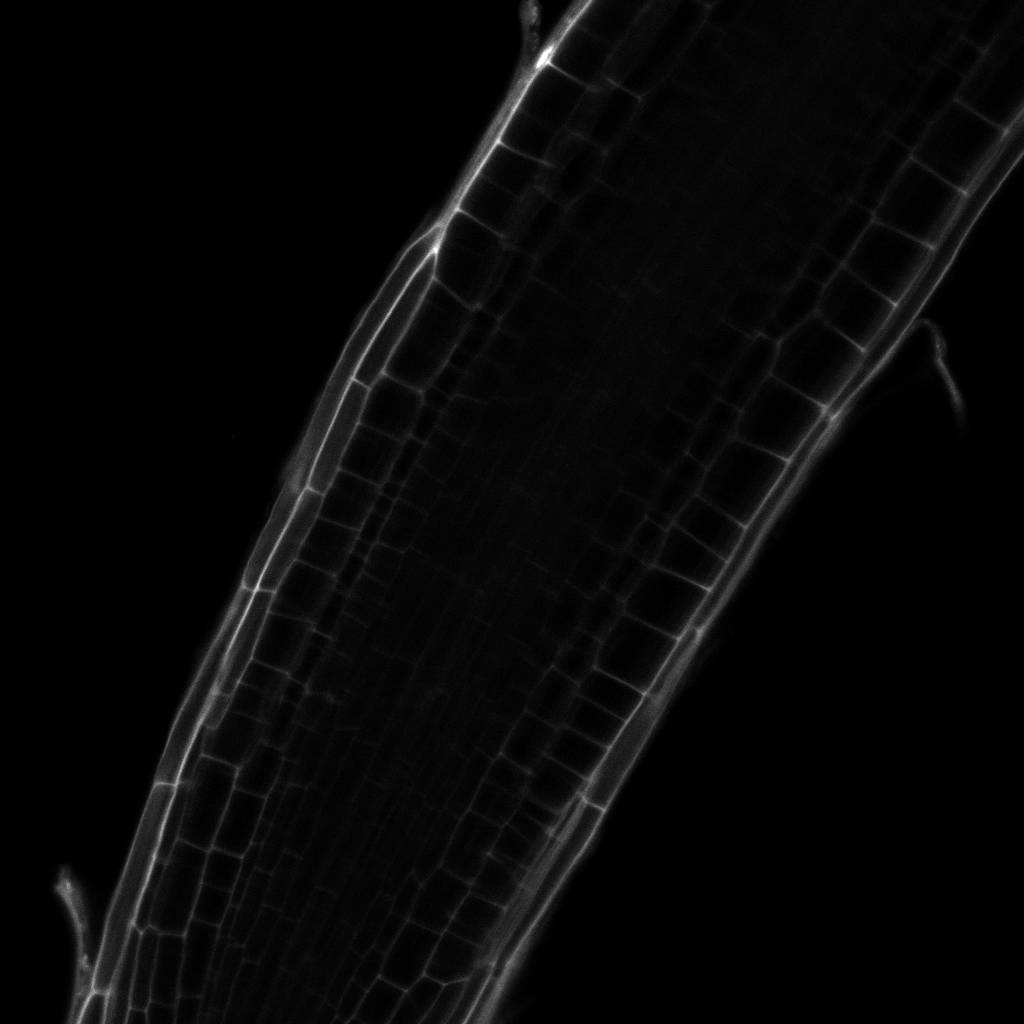

Supplement: Supplementary file 5 — Source data Fig. 4 [file 44319_2026_737_MOESM5_ESM.zip › Figure 4/4E/WT_BL_1.tif]

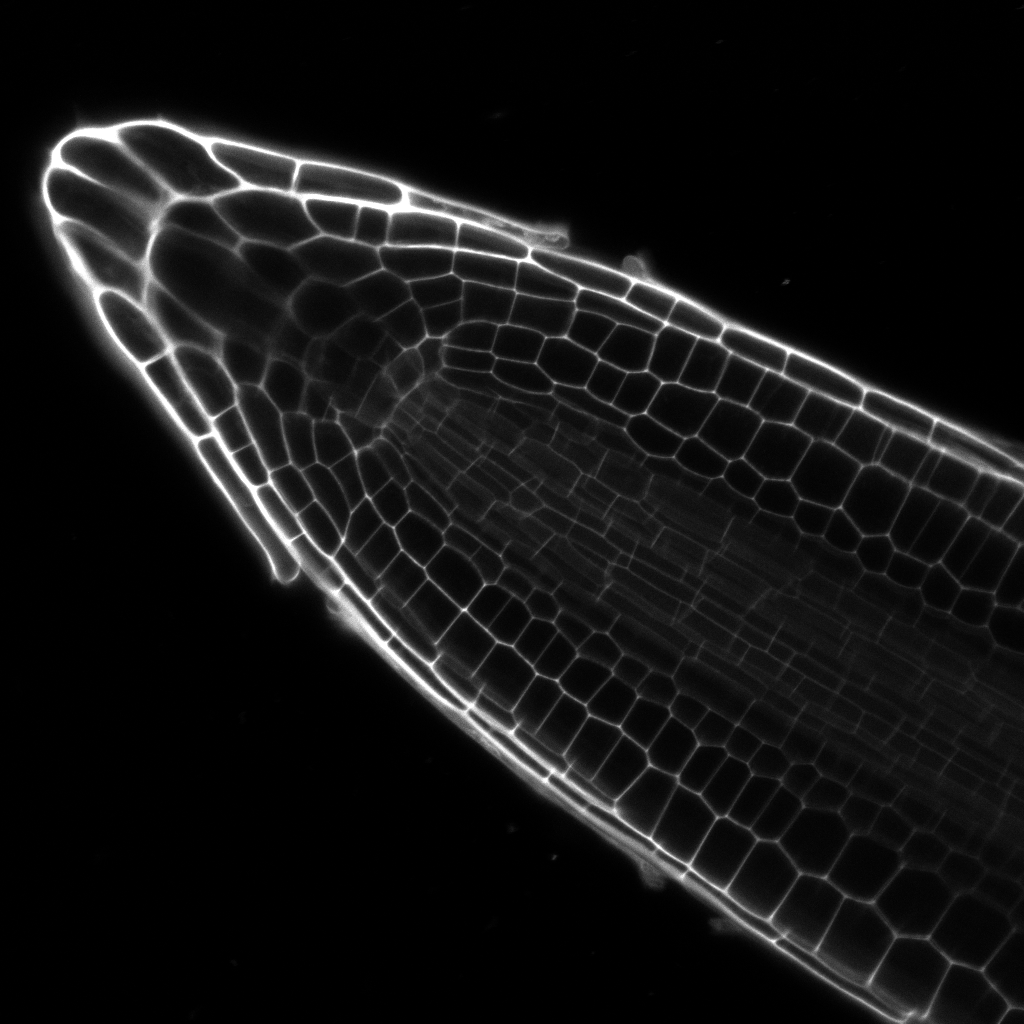

Supplement: Supplementary file 5 — Source data Fig. 4 [file 44319_2026_737_MOESM5_ESM.zip › Figure 4/4E/cycd3;3_CTRL.tif]

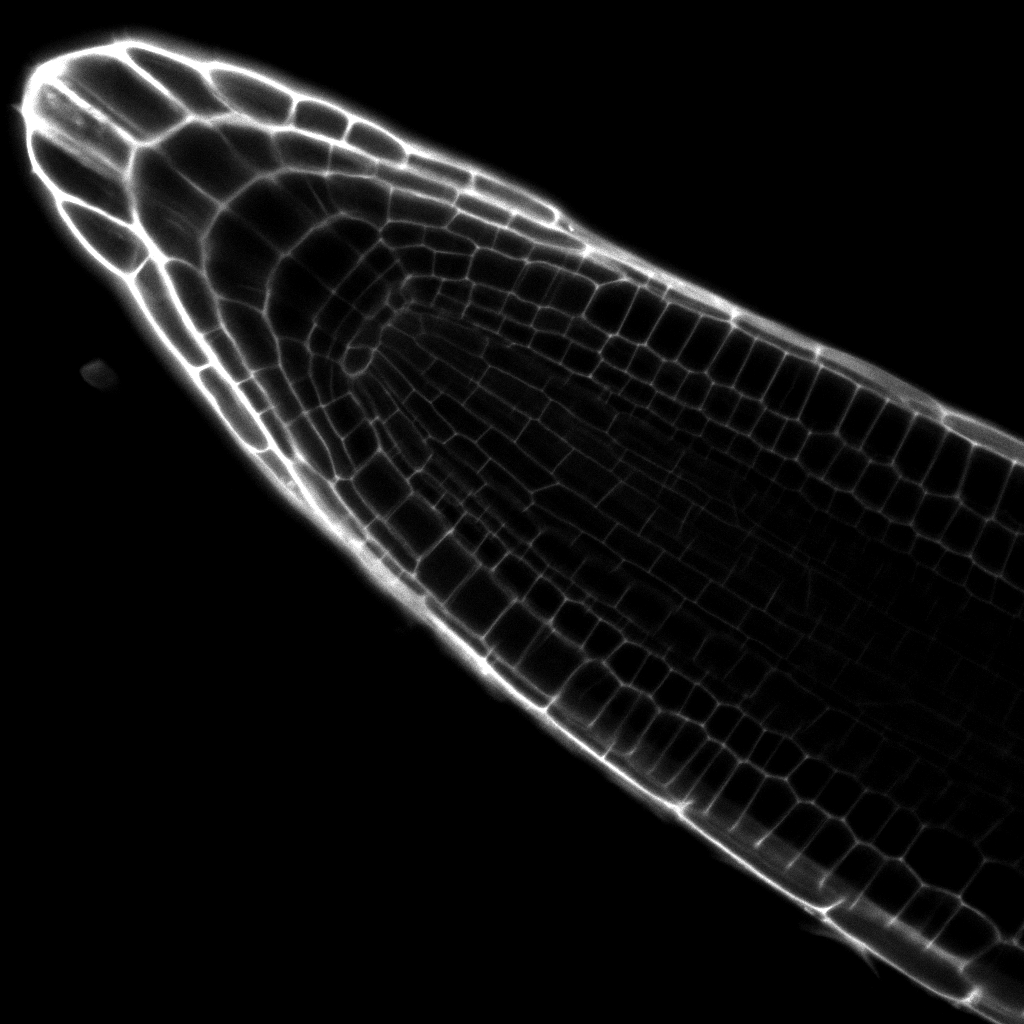

Supplement: Supplementary file 5 — Source data Fig. 4 [file 44319_2026_737_MOESM5_ESM.zip › Figure 4/4E/WT_CTRL.tif]

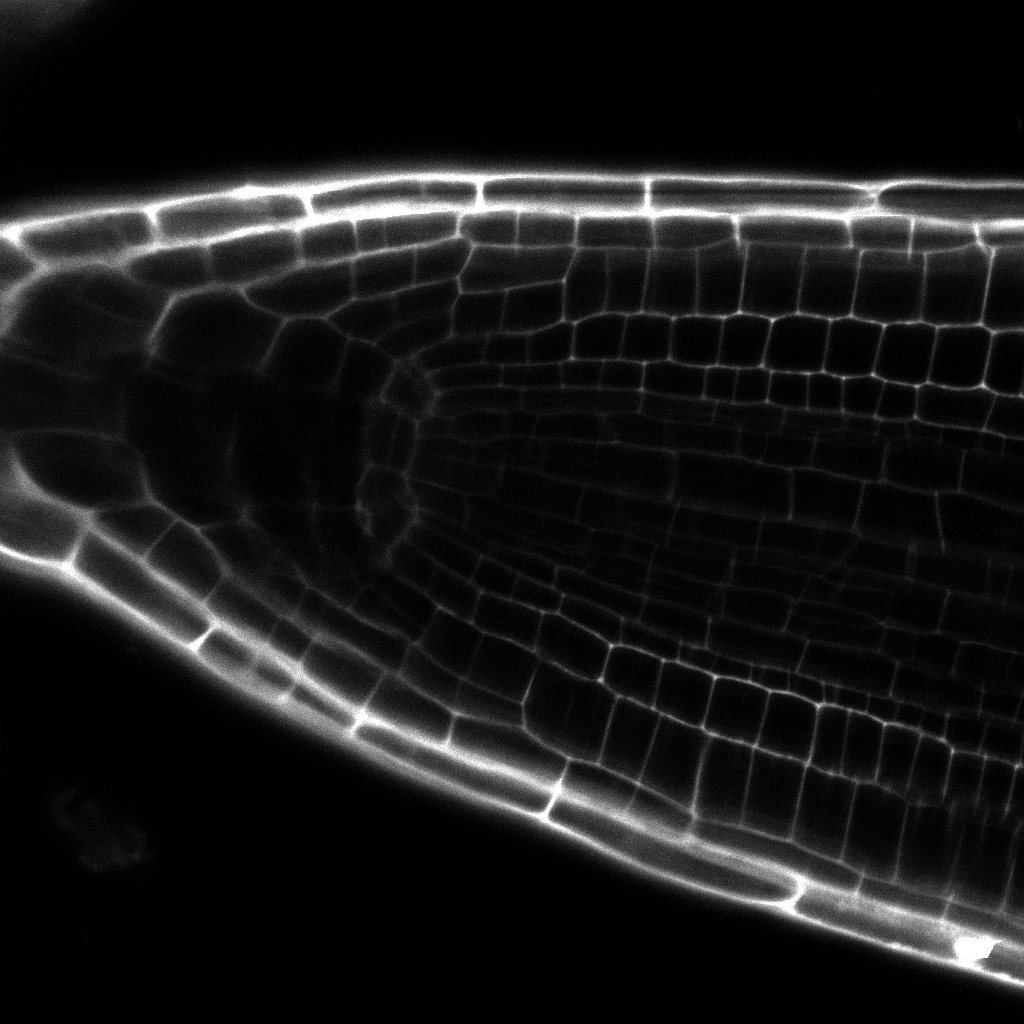

Supplement: Supplementary file 5 — Source data Fig. 4 [file 44319_2026_737_MOESM5_ESM.zip › Figure 4/4G/cycd3;3.tif]

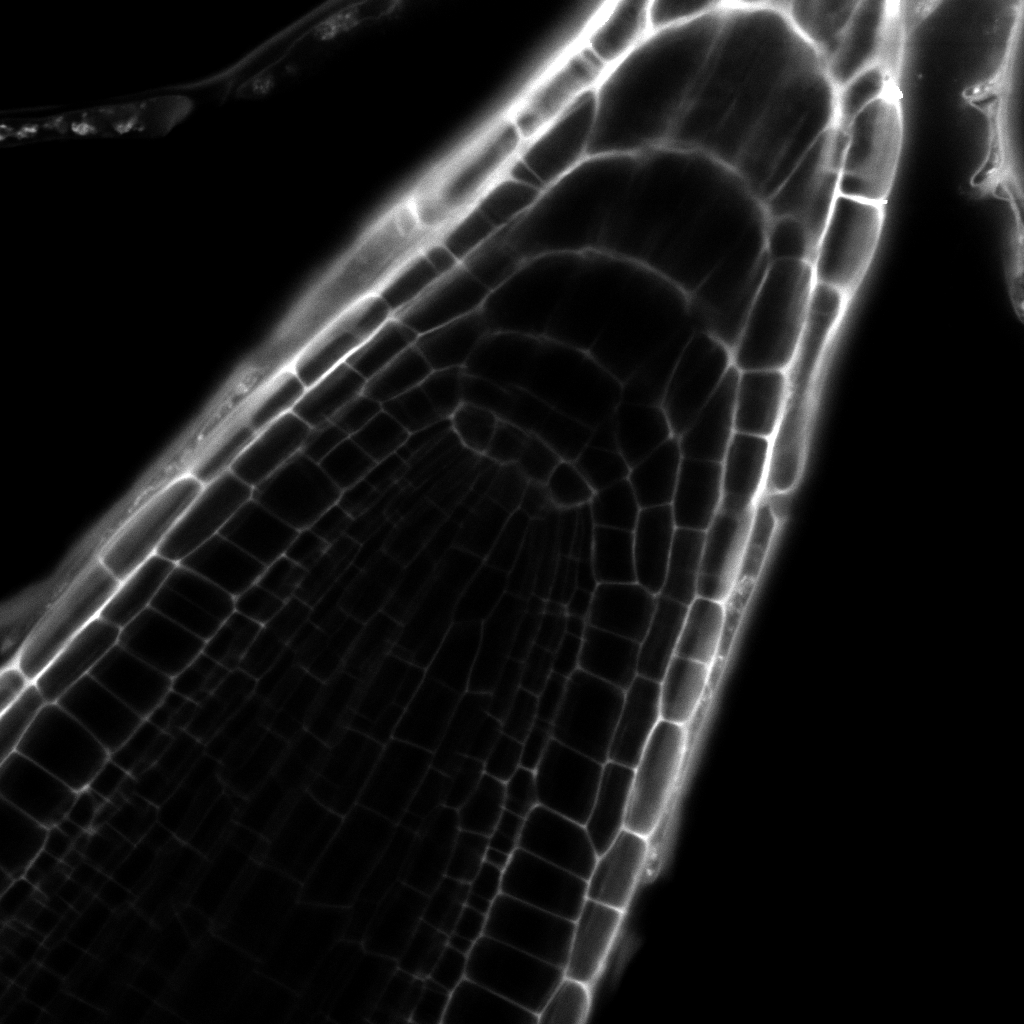

Supplement: Supplementary file 5 — Source data Fig. 4 [file 44319_2026_737_MOESM5_ESM.zip › Figure 4/4G/WT.tif]

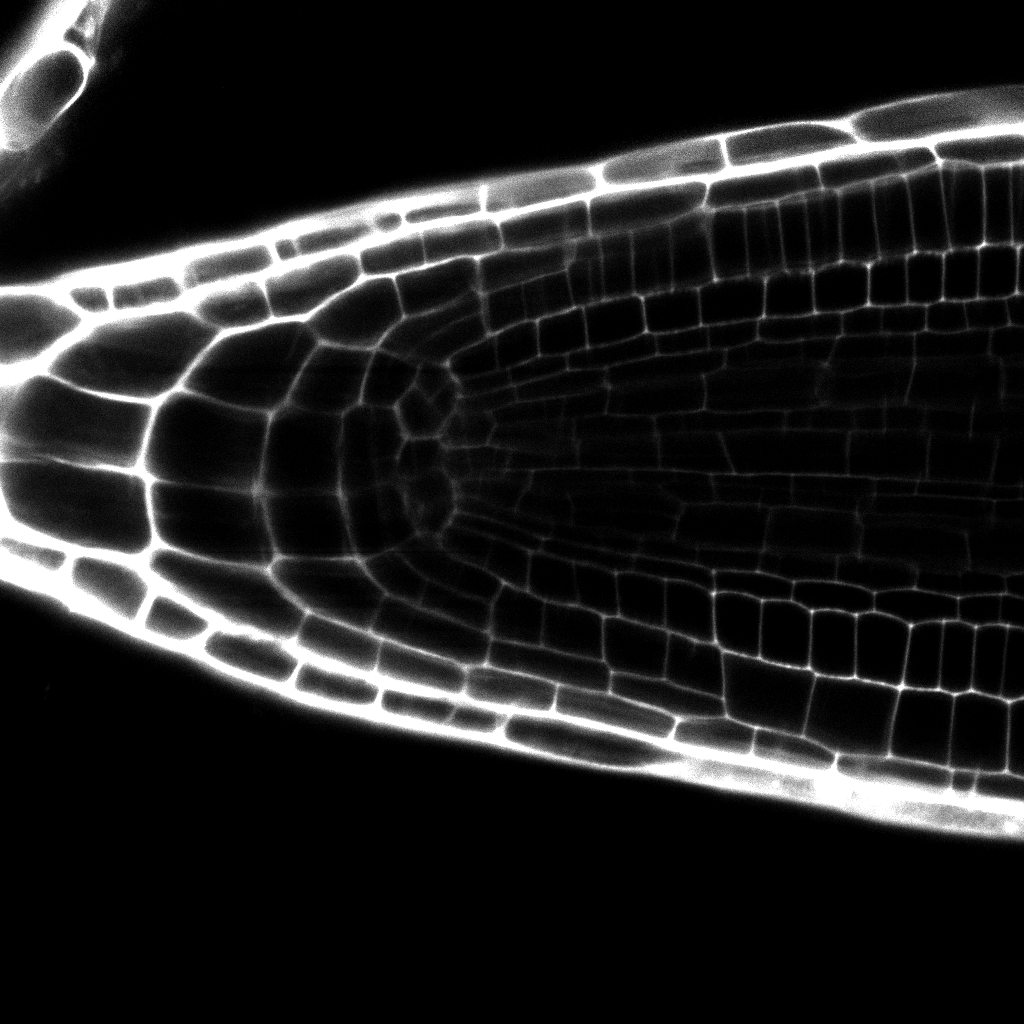

Supplement: Supplementary file 5 — Source data Fig. 4 [file 44319_2026_737_MOESM5_ESM.zip › Figure 4/4G/cycd3;3 bzr1-2.tif]

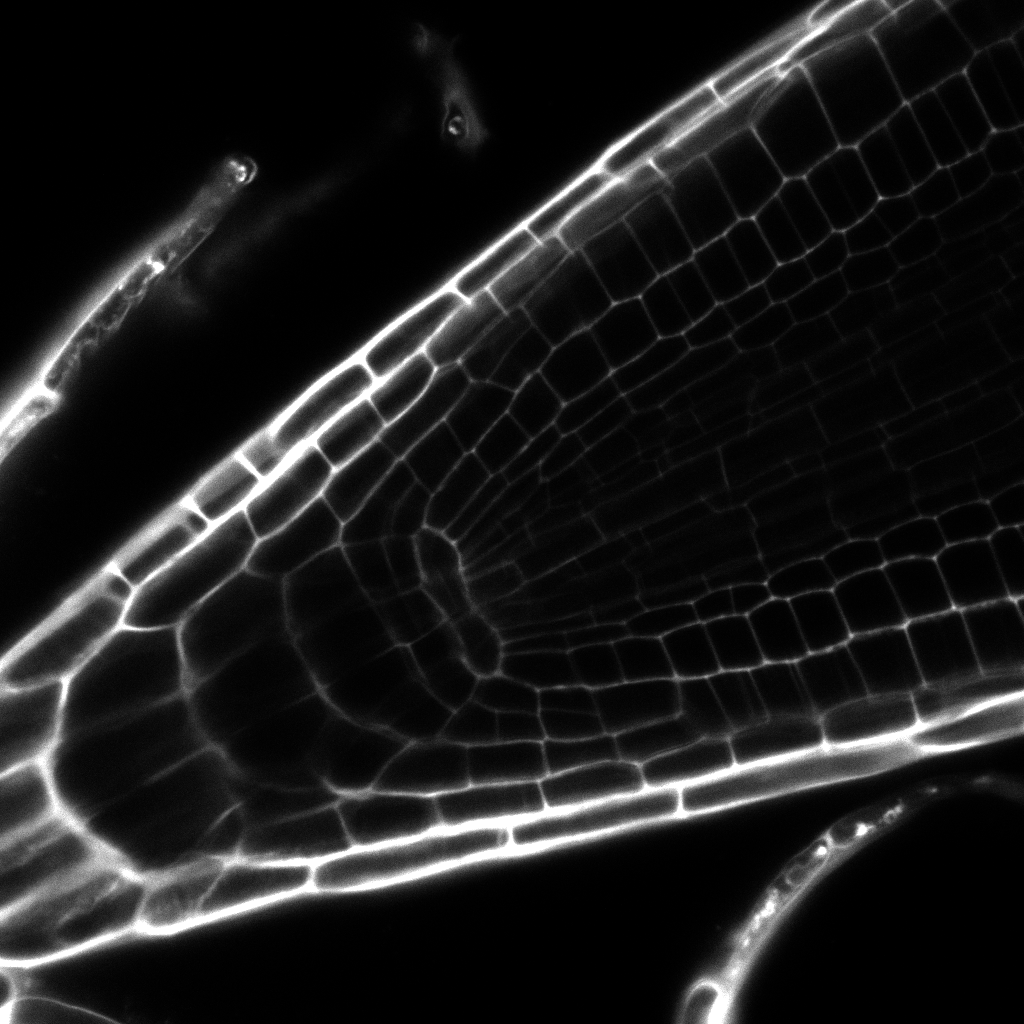

Supplement: Supplementary file 5 — Source data Fig. 4 [file 44319_2026_737_MOESM5_ESM.zip › Figure 4/4G/bzr1-2.tif]

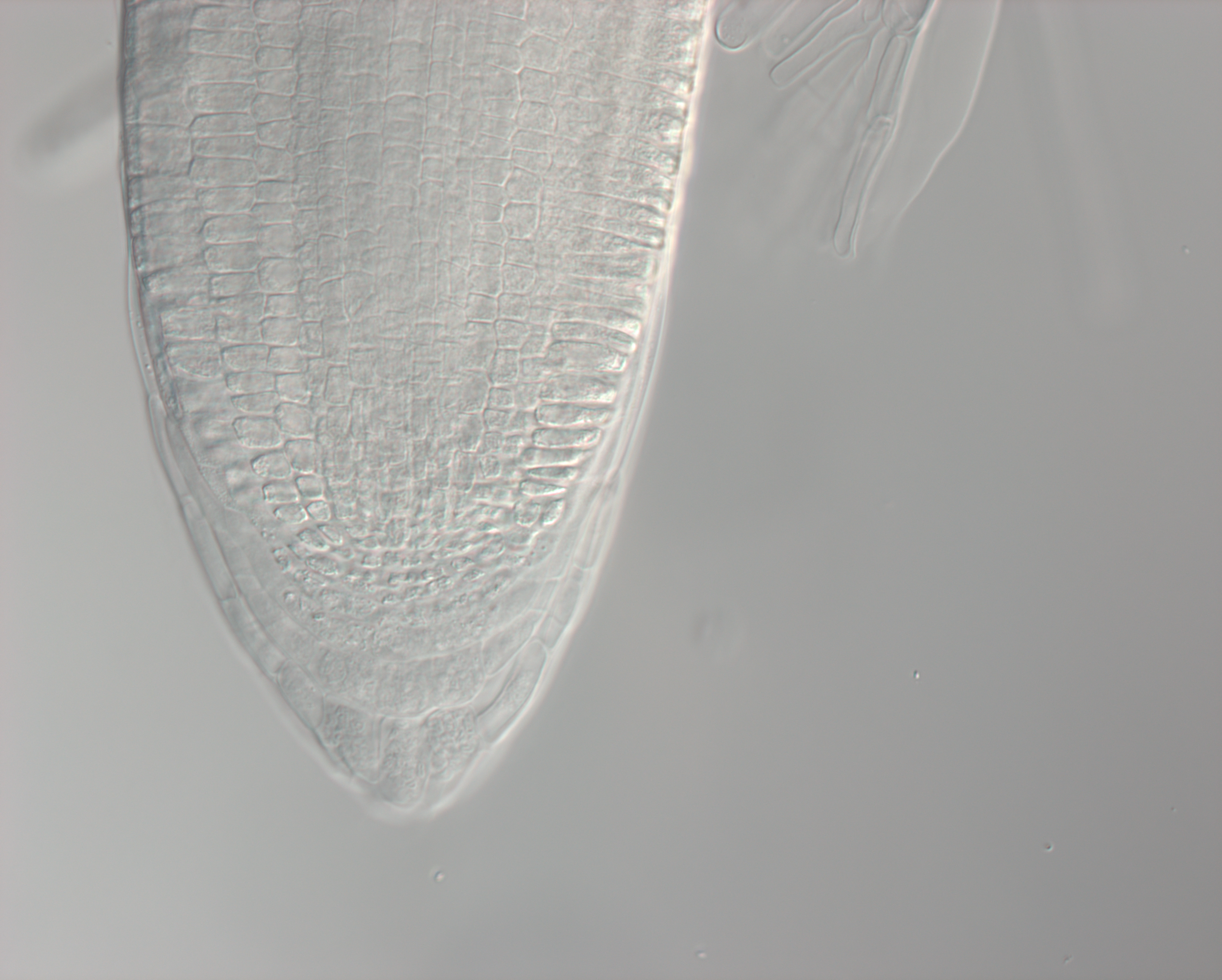

Supplement: Supplementary file 6 — EV Figures Source Data [file 44319_2026_737_MOESM6_ESM.zip › Figures EV/Figure EV 1/1A/det2-1_1.png]

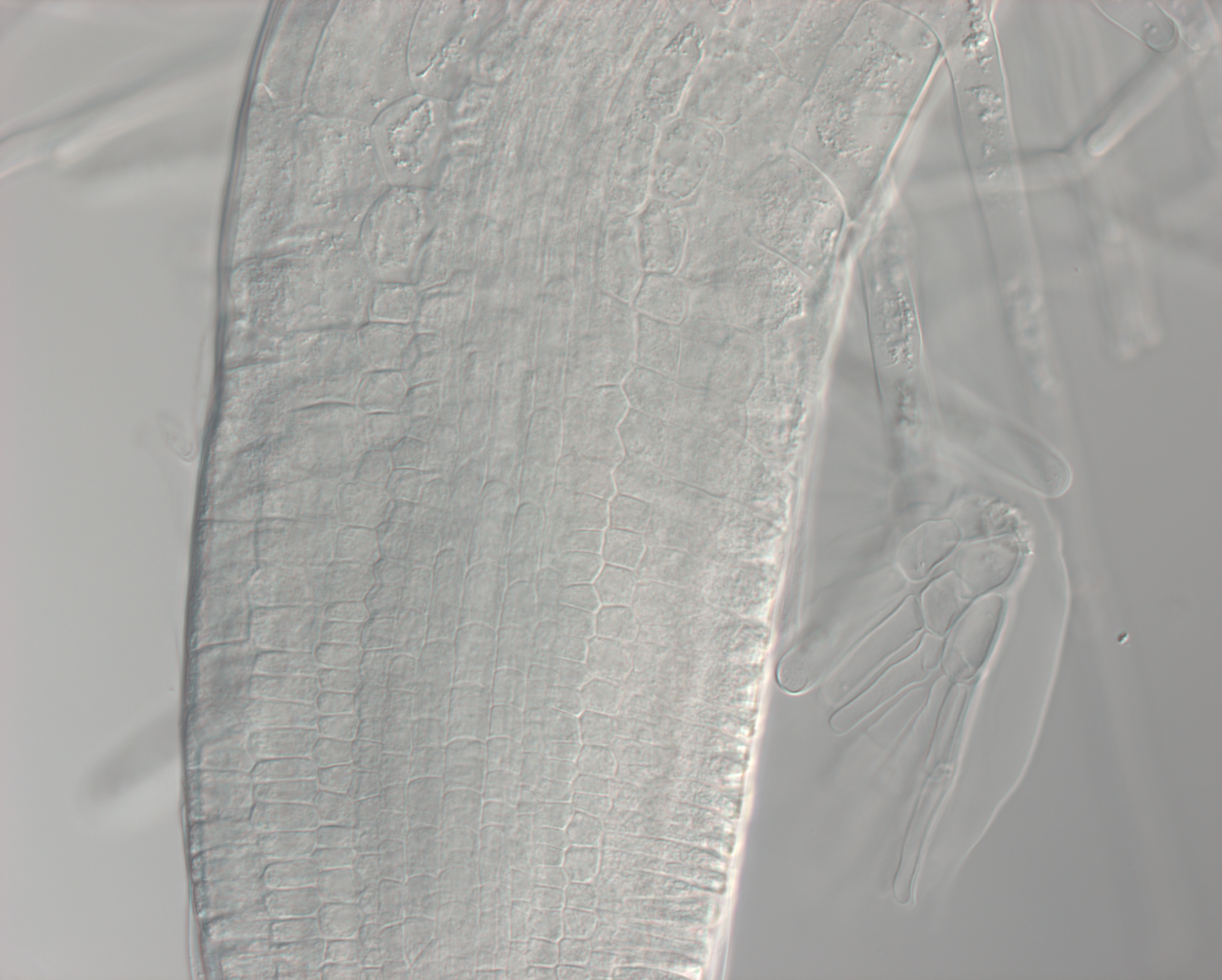

Supplement: Supplementary file 6 — EV Figures Source Data [file 44319_2026_737_MOESM6_ESM.zip › Figures EV/Figure EV 1/1A/det2-1_2.png]

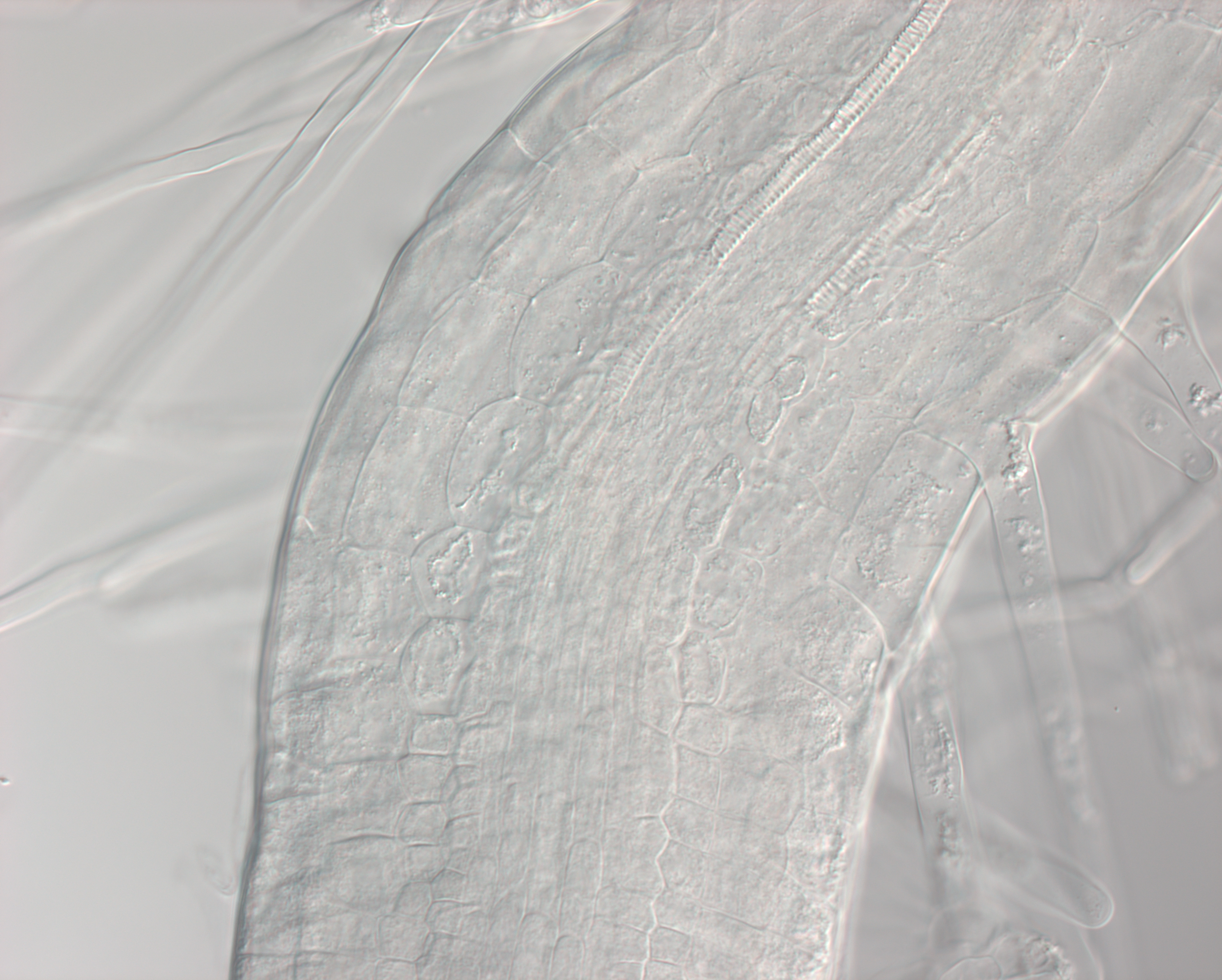

Supplement: Supplementary file 6 — EV Figures Source Data [file 44319_2026_737_MOESM6_ESM.zip › Figures EV/Figure EV 1/1A/det2-1_3.png]

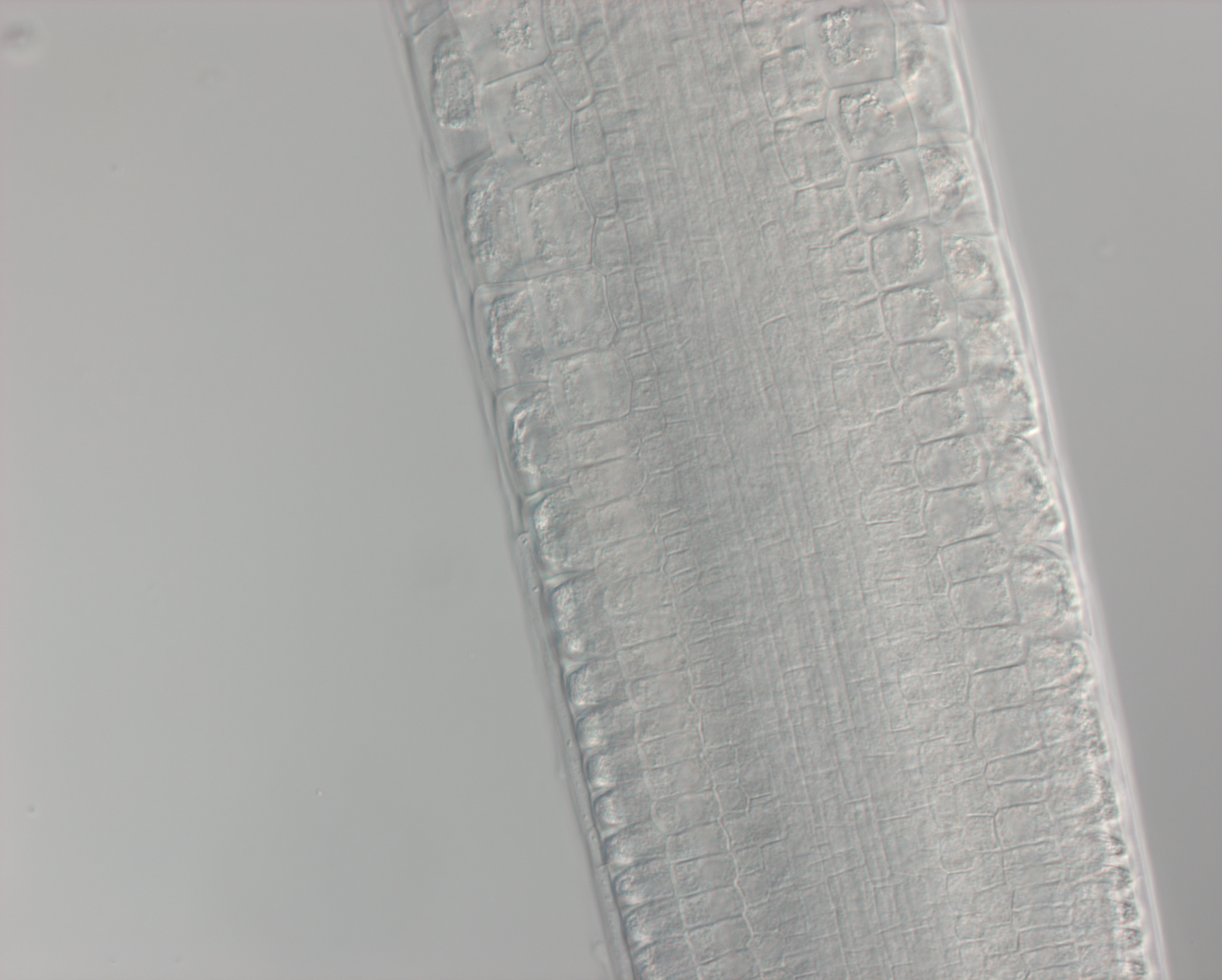

Supplement: Supplementary file 6 — EV Figures Source Data [file 44319_2026_737_MOESM6_ESM.zip › Figures EV/Figure EV 1/1A/WT_3.png]

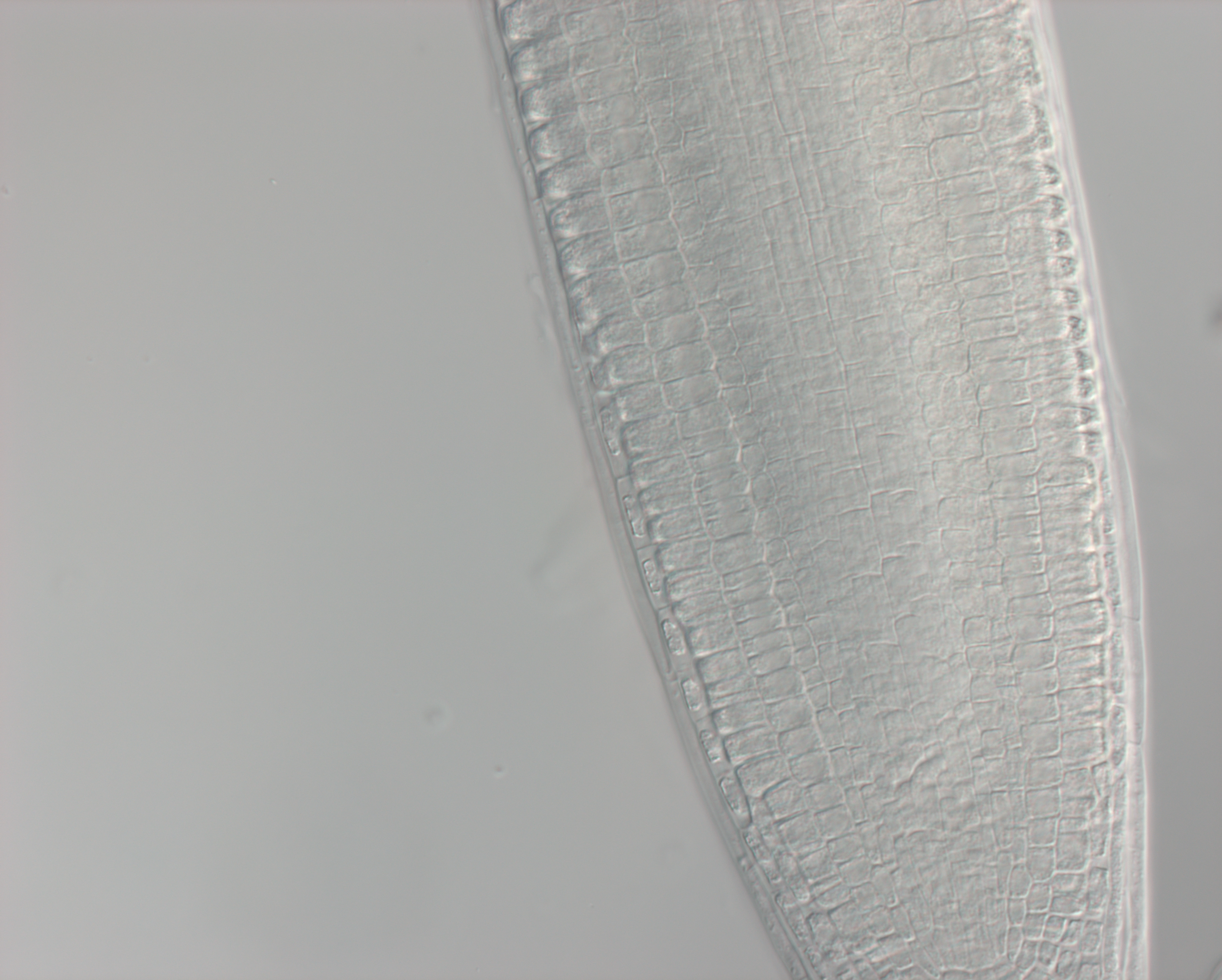

Supplement: Supplementary file 6 — EV Figures Source Data [file 44319_2026_737_MOESM6_ESM.zip › Figures EV/Figure EV 1/1A/WT_2.png]

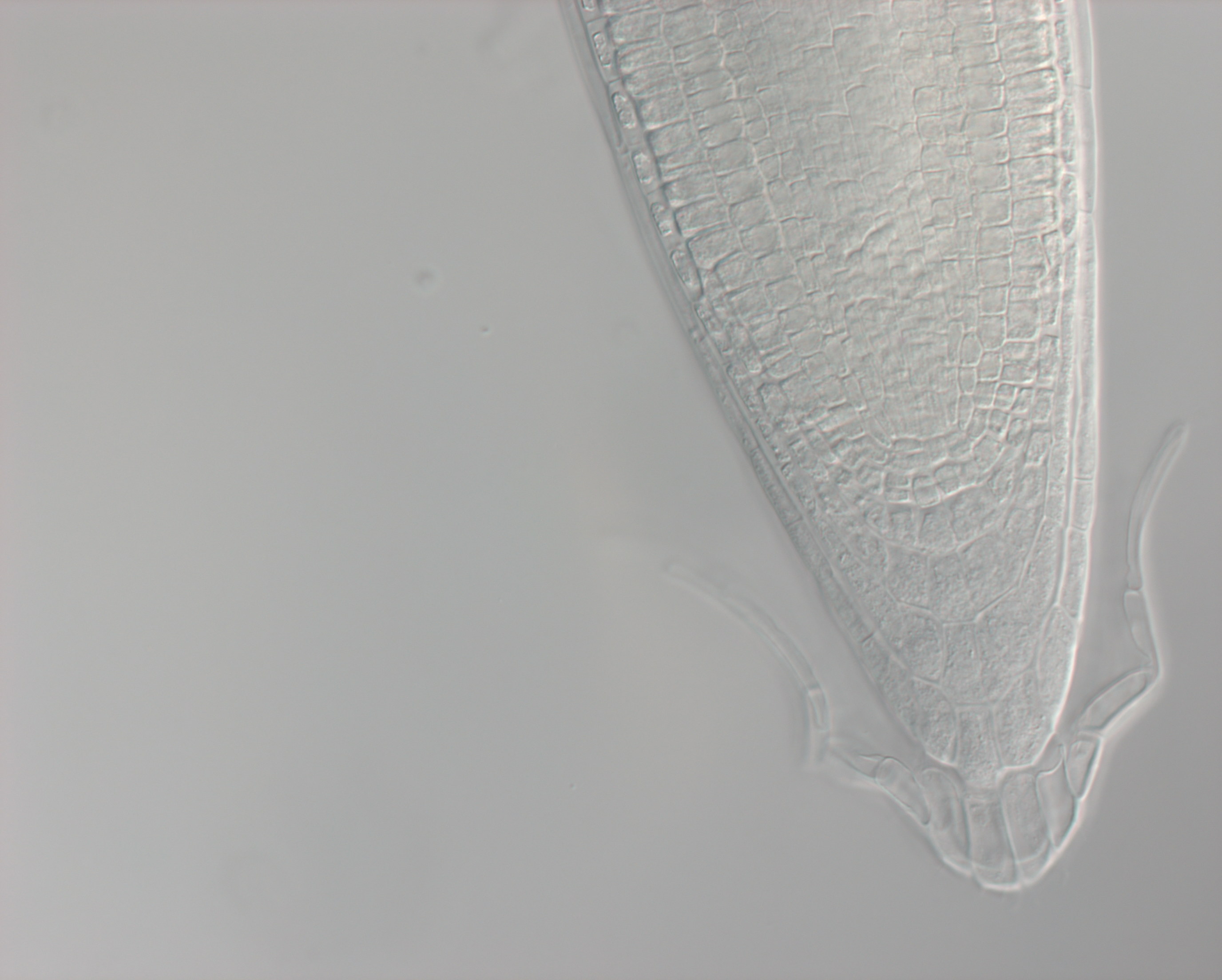

Supplement: Supplementary file 6 — EV Figures Source Data [file 44319_2026_737_MOESM6_ESM.zip › Figures EV/Figure EV 1/1A/WT_1.png]

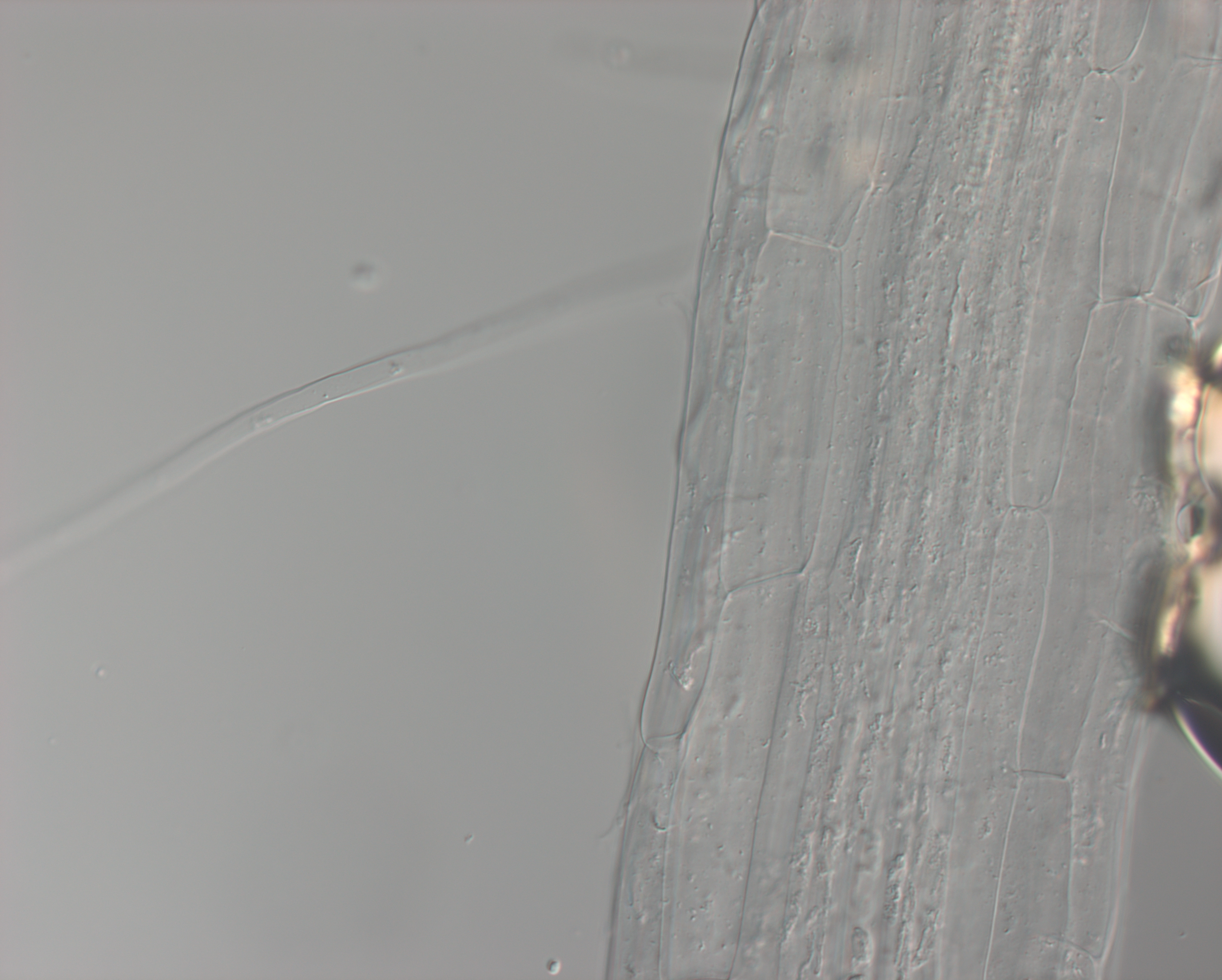

Supplement: Supplementary file 6 — EV Figures Source Data [file 44319_2026_737_MOESM6_ESM.zip › Figures EV/Figure EV 1/1A/35S ask theta_3.png]

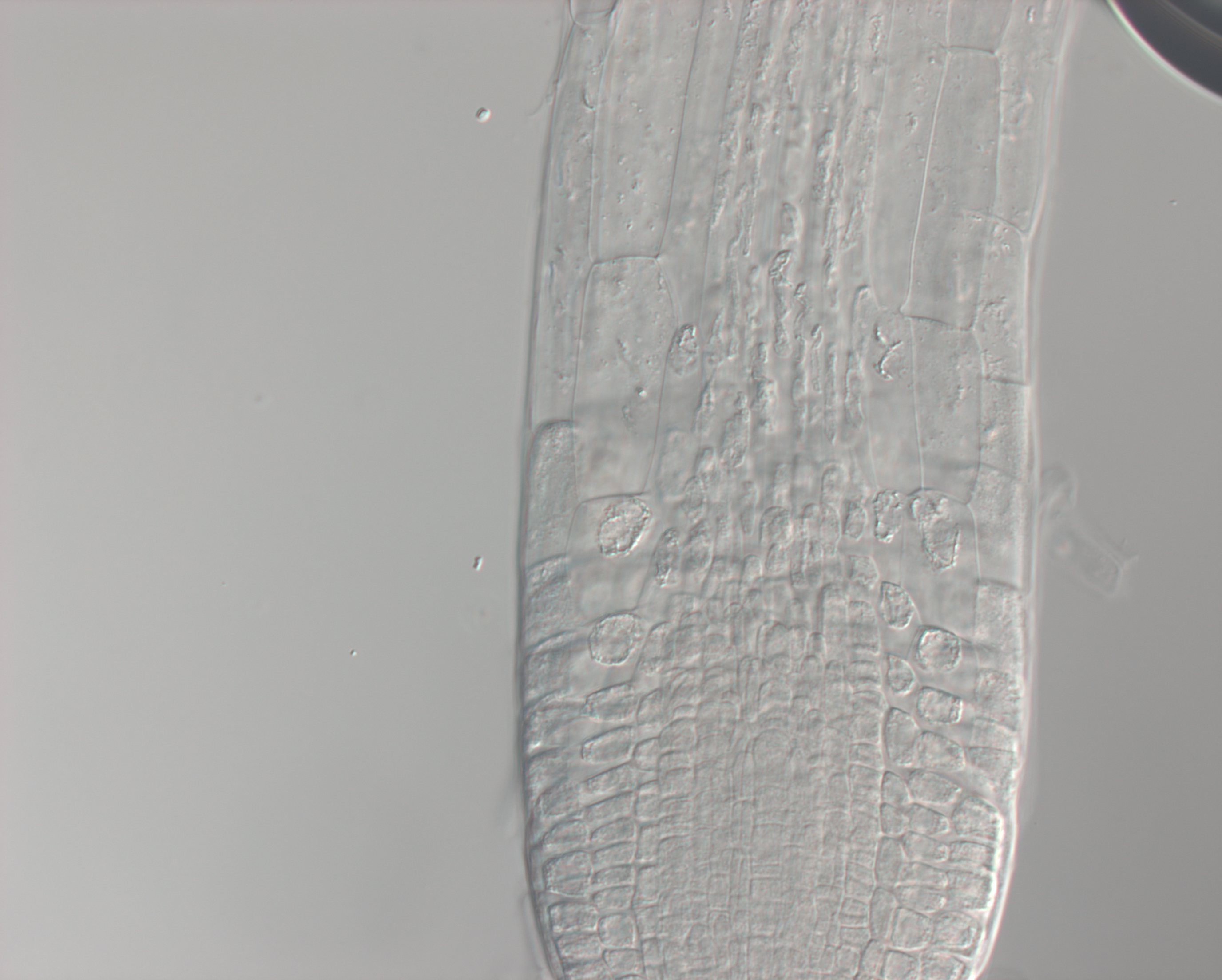

Supplement: Supplementary file 6 — EV Figures Source Data [file 44319_2026_737_MOESM6_ESM.zip › Figures EV/Figure EV 1/1A/35S ask theta_2.png]

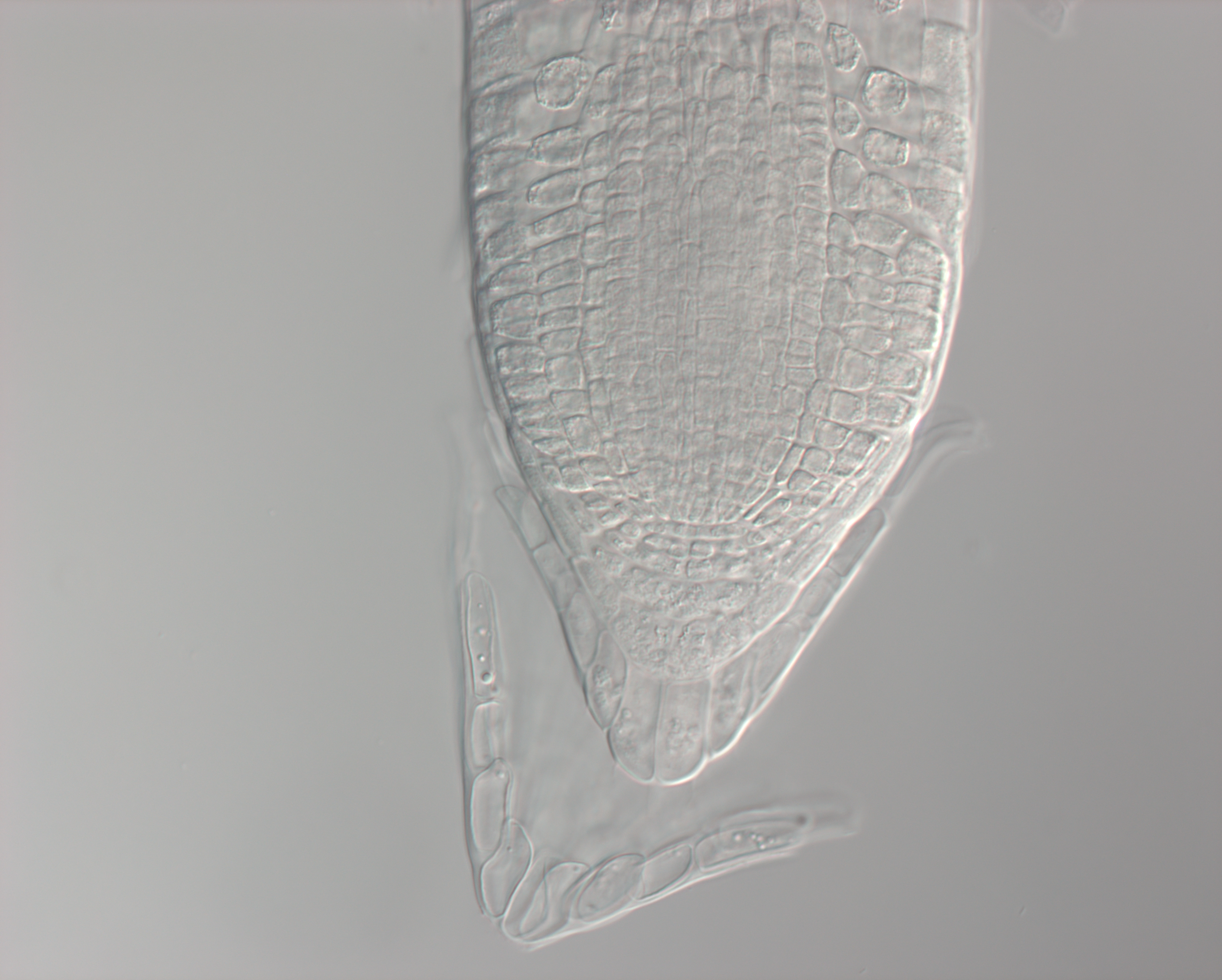

Supplement: Supplementary file 6 — EV Figures Source Data [file 44319_2026_737_MOESM6_ESM.zip › Figures EV/Figure EV 1/1A/35S ask theta_1.png]

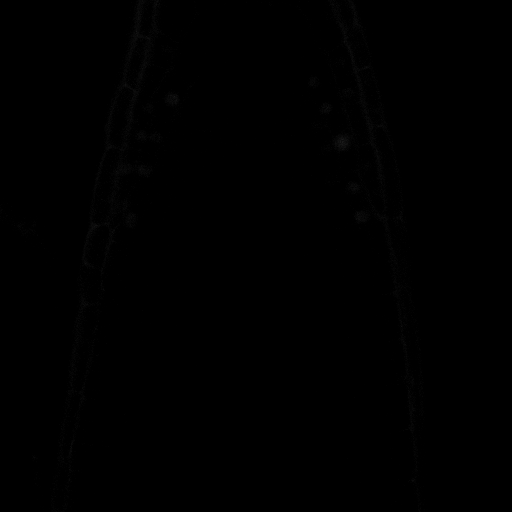

Supplement: Supplementary file 6 — EV Figures Source Data [file 44319_2026_737_MOESM6_ESM.zip › Figures EV/Figure EV3/3H/+DEX.tif]

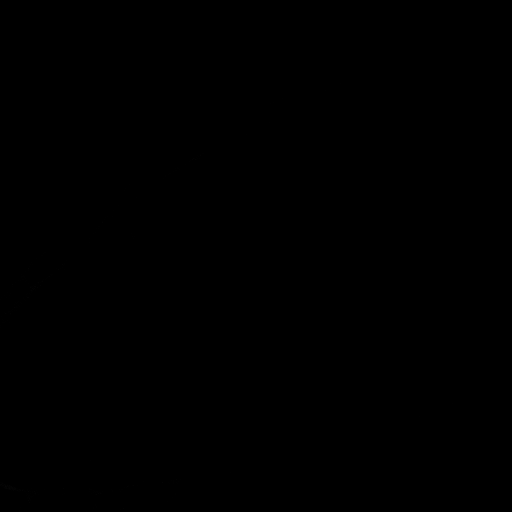

Supplement: Supplementary file 6 — EV Figures Source Data [file 44319_2026_737_MOESM6_ESM.zip › Figures EV/Figure EV3/3H/CTRL.tif]

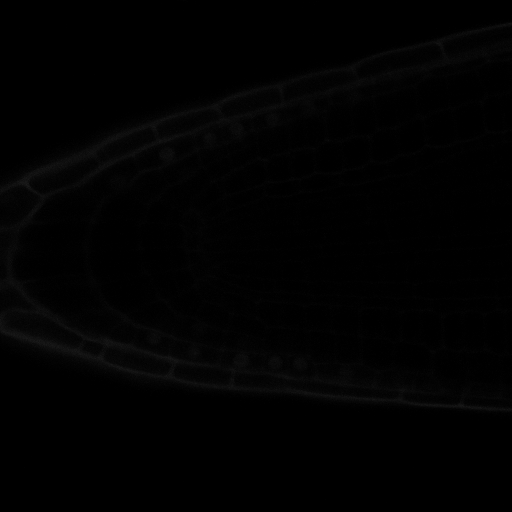

Supplement: Supplementary file 6 — EV Figures Source Data [file 44319_2026_737_MOESM6_ESM.zip › Figures EV/Figure EV3/3F/+DEX.tif]

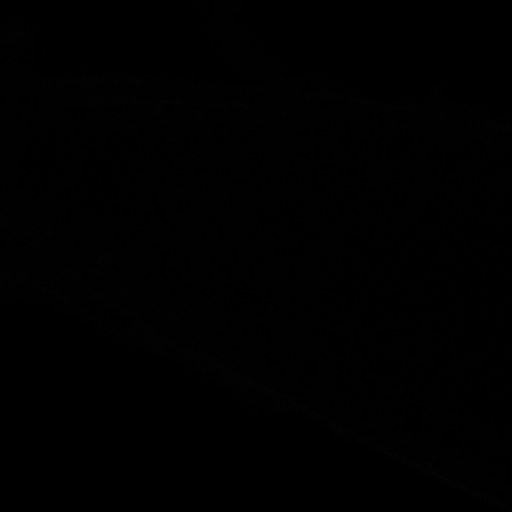

Supplement: Supplementary file 6 — EV Figures Source Data [file 44319_2026_737_MOESM6_ESM.zip › Figures EV/Figure EV3/3F/CTRL.tif]

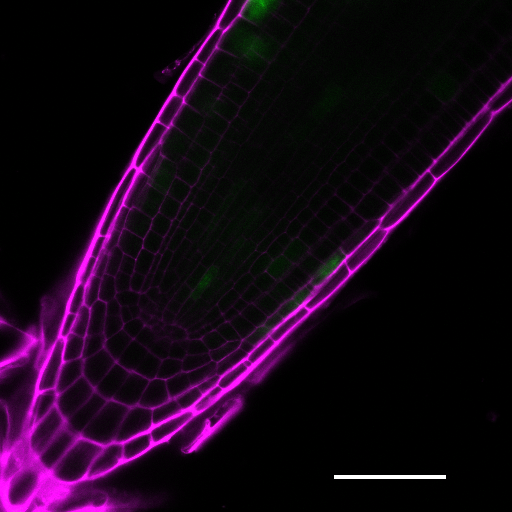

Supplement: Supplementary file 6 — EV Figures Source Data [file 44319_2026_737_MOESM6_ESM.zip › Figures EV/Figure EV2/2C/+BL.png]
