# Supplementary material for: Brassinosteroids control cell proliferation in the lateral root cap of the Arabidopsis root
Source: EMBO Rep. 2026 Apr 10;27(9):2183–200. doi: 10.1038/s44319-026-00737-0 (PMC13172465; doi:10.1038/s44319-026-00737-0)
Supplement: Supplementary file 3 — Source data Fig. 2 [file 44319_2026_737_MOESM3_ESM.zip › Figure 2/2A/READEME.rtf]

Overlapping  confocal images of root tip from the indicated lines. Overlapping tiles are labeled sequentially name_1, name_2 etc. 
