# Supplementary material for: Brassinosteroids control cell proliferation in the lateral root cap of the Arabidopsis root
Source: EMBO Rep. 2026 Apr 10;27(9):2183–200. doi: 10.1038/s44319-026-00737-0 (PMC13172465; doi:10.1038/s44319-026-00737-0)
Supplement: Supplementary file 3 — Source data Fig. 2 [file 44319_2026_737_MOESM3_ESM.zip › Figure 2/2C/README.rtf]

Confocal images from root tips of J3411 plants treated with control, +BL and +PPZ as indicated in the image file name. 
