# Supplementary material for: Brassinosteroids control cell proliferation in the lateral root cap of the Arabidopsis root
Source: EMBO Rep. 2026 Apr 10;27(9):2183–200. doi: 10.1038/s44319-026-00737-0 (PMC13172465; doi:10.1038/s44319-026-00737-0)
Supplement: Supplementary file 4 — Source data Fig. 3 [file 44319_2026_737_MOESM4_ESM.zip › Figure 3/3G/README3g.rtf]

Maximum projections of z-stack confocal images from the Timer-NLS roots in the WT or bzr1-2 genetic background as indicated in the file name.
