# Supplementary material for: Brassinosteroids control cell proliferation in the lateral root cap of the Arabidopsis root
Source: EMBO Rep. 2026 Apr 10;27(9):2183–200. doi: 10.1038/s44319-026-00737-0 (PMC13172465; doi:10.1038/s44319-026-00737-0)
Supplement: Supplementary file 5 — Source data Fig. 4 [file 44319_2026_737_MOESM5_ESM.zip › Figure 4/4E/README.rtf]

Confocal images from roots of the indicated lines, after control (indicated as name_CTRL) or BL treatment (name_BL).  Overlapping tiles are labeled sequentially name_1, name_2.
