# Supplementary material for: Brassinosteroids control cell proliferation in the lateral root cap of the Arabidopsis root
Source: EMBO Rep. 2026 Apr 10;27(9):2183–200. doi: 10.1038/s44319-026-00737-0 (PMC13172465; doi:10.1038/s44319-026-00737-0)
Supplement: Supplementary file 6 — EV Figures Source Data [file 44319_2026_737_MOESM6_ESM.zip › Figures EV/Figure EV4/4E/README.rtf]

Maximum projections of z-stack confocal images of roots from the F1 progeny form pFEZ>bzr1-D>citNLS x pCYCD3;3::GFP. CTRL treatment or DEX treatment are indicated in the file name. 
