# Supplementary material for: Brassinosteroids control cell proliferation in the lateral root cap of the Arabidopsis root
Source: EMBO Rep. 2026 Apr 10;27(9):2183–200. doi: 10.1038/s44319-026-00737-0 (PMC13172465; doi:10.1038/s44319-026-00737-0)
Supplement: Supplementary file 6 — EV Figures Source Data [file 44319_2026_737_MOESM6_ESM.zip › Figures EV/Figure EV4/4G/README.rtf]

Confocal images of pCYCD3;3::GFP plants in the WT or bzr1-2 background as indicated in the file name. 
