# Supplementary material for: Brassinosteroids control cell proliferation in the lateral root cap of the Arabidopsis root
Source: EMBO Rep. 2026 Apr 10;27(9):2183–200. doi: 10.1038/s44319-026-00737-0 (PMC13172465; doi:10.1038/s44319-026-00737-0)
Supplement: Supplementary file 6 — EV Figures Source Data [file 44319_2026_737_MOESM6_ESM.zip › Figures EV/Figure EV3/3J/README.rtf]

Confocal images of roots from the indicated lines (file name). Tiled stack images are indicated as name_1 and name_2. 
