# Supplementary material for: Brassinosteroids control cell proliferation in the lateral root cap of the Arabidopsis root
Source: EMBO Rep. 2026 Apr 10;27(9):2183–200. doi: 10.1038/s44319-026-00737-0 (PMC13172465; doi:10.1038/s44319-026-00737-0)
Supplement: Supplementary file 7 — Expanded View Figures [file 44319_2026_737_MOESM7_ESM.pdf]

Expanded View Figures

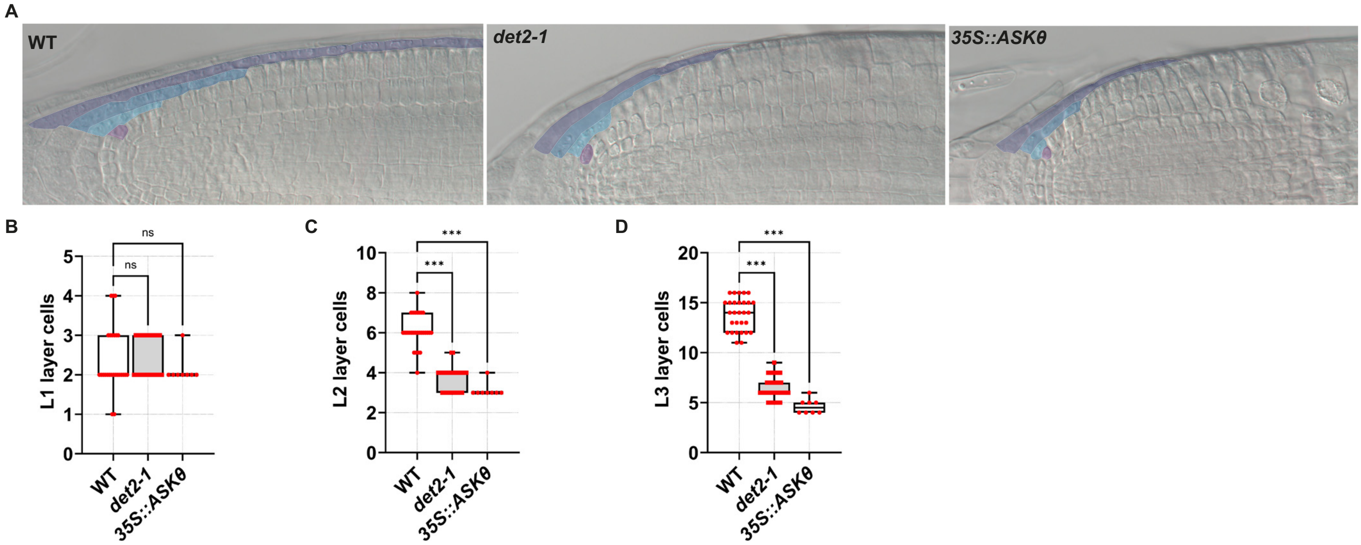

Figure EV1. Defects in the BR biosynthesis and signalling affect LRC layer size.

(**A**) Representative images of cleared root tips of wild-type (Col-0), *det2-1* and *35S::ASKθ* plants at 6 dpg. LRC layers and the stem cell are artificially coloured corresponding to the colours in (**A**). (**B–D**) Box plots showing the quantification of the L1 layer cells (**B**), L2 layer cells (**C**) and L3 layer cells (**D**) in wild-type (L1 $n = 26$, L2 $n = 28$, L3 $n = 29$), *det2-1* (L1 $n = 30$, L2 $n = 38$, L3 n = 38) and *35S::ASKθ* (L1-L3 $n = 8$) plants at 6 dpg. Statistical differences between the number of cells per layer were calculated with the nonparametric Kruskal–Wallis test, followed by Dunn's multiple-comparisons post hoc test for pairwise comparisons (adjusted P values): L1 cells in (**B**) WT vs. *det2-1*, ns$P = 0.78$; and WT vs. *35S::ASKθ*, ns (P = 0.66); L2 cells in (**C**) WT vs. *det2-1*, ***$P < 0.001$; and WT vs. *35S::ASKθ*, ***$P < 0.001$); L3 cells in (**D**) WT vs. *det2-1*, ***$P < 0.001$; and WT vs. *35S::ASKθ*, ***$P < 0.001$. Scale bars indicate 50 µm. Box plots show the median (centre line), with the box spanning the first to third quartiles (Q1–Q3); whiskers indicate the minimum (Q0) and maximum (Q4) values. Source data are available online for this figure.

 © The Author(s)

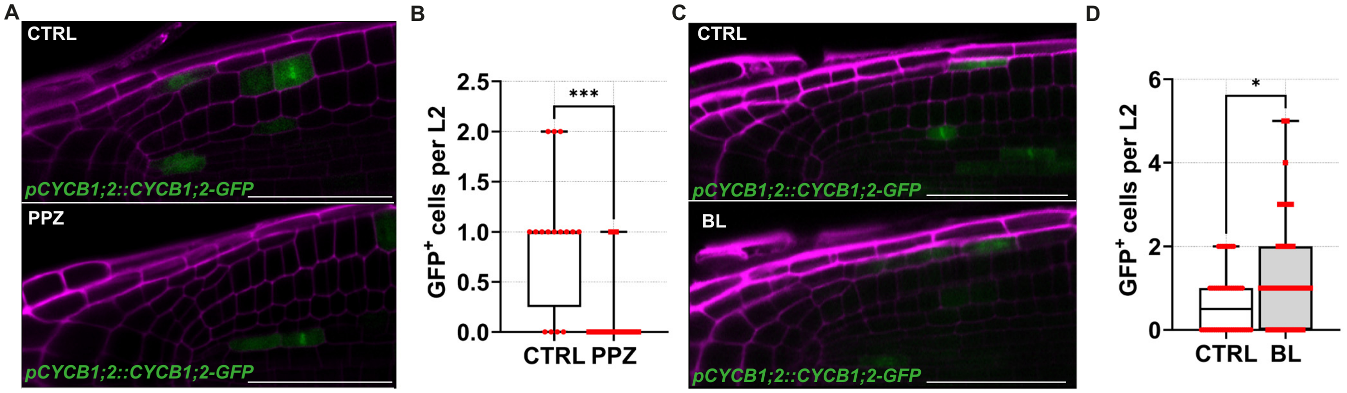

Figure EV2. BR promote mitotic cell divisions in the L2 layer.

(**A**) Z-stack projection of representative images of the root cap in *pCYCB1;2::CYCB1;2-GFP* plants at 6 dpg after a 24-h mock (CTRL) or 2 µM propiconazole (PPZ) treatment. (**B**) Box plots showing the quantification of CYCB1;2-GFP positive cells per L2 layer in treatments from (**A**). CTRL ($n = 16$) and PPZ ($n = 22$). Pairwise comparison of the CYCB1;2-GFP positive (GFP^+) cells between CTRL and PPZ was analysed using the nonparametric two-tailed Mann–Whitney *U* test (exact *P* value): CTRL vs. PPZ, ***$P < 0.001$. (**C**) Z-stack projection of representative images of the root cap in *pCYCB1;2::CYCB1;2-GFP* plants at 6 dpg after 6-h mock (CTRL) or 1 nM epibrassinolide (BL) treatment. (**D**) Box plots showing the quantification of the CYCB1;2-GFP positive cells per L2 layer in treatments from (**C**). CTRL ($n = 48$) and BL ($n = 45$). Pairwise comparison of the CYCB1;2-GFP positive (GFP^+) cells between CTRL and BL was analysed using the nonparametric two-tailed Mann–Whitney *U* test (exact *P* value): CTRL vs. BL, *$P = 0.01$. Scale bars = 50 µm. Box plots show the median (centre line), with the box spanning the first to third quartiles (Q1–Q3); whiskers indicate the minimum (Q0) and maximum (Q4) values. Source data are available online for this figure.

© The Author(s)

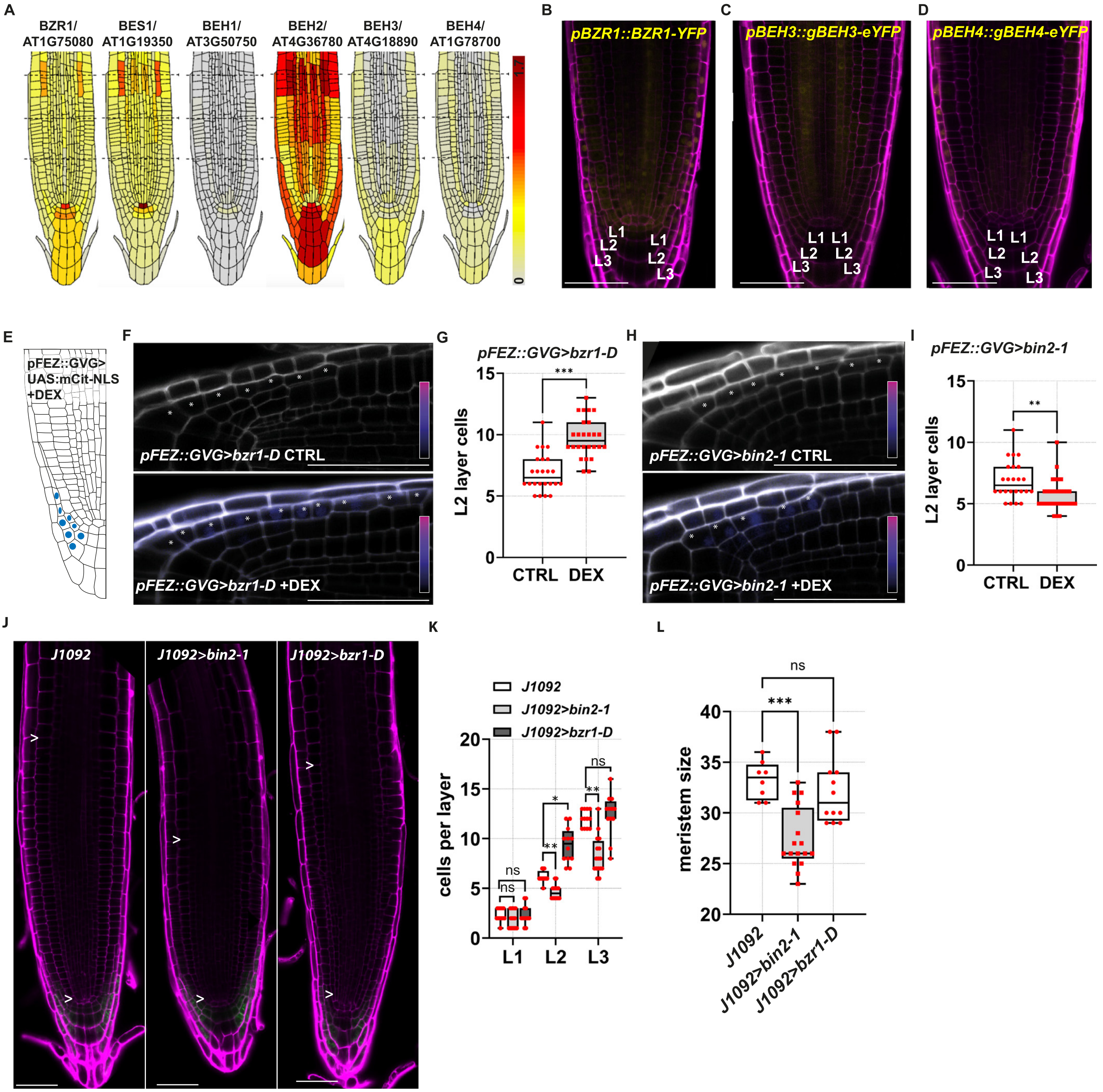
A
BZR1/ AT1G75080
BES1/ AT1G19350
BEH1/ AT3G50750
BEH2/ AT4G36780
BEH3/ AT4G18890
BEH4/ AT1G78700
0
B
pBZR1::BZR1-YFP
C
pBEH3::gBEH3-eYFP
D
pBEH4::gBEH4-eYFP
L1 L2 L3
E
pFEZ::GVG> UAS:mCit-NLS +DEX
F
pFEZ::GVG>bzr1-D CTRL
pFEZ::GVG>bzr1-D +DEX
G
pFEZ::GVG>bzr1-D
L2 layer cells
CTRL DEX
***
H
pFEZ::GVG>bin2-1 CTRL
pFEZ::GVG>bin2-1 +DEX
I
pFEZ::GVG>bin2-1
L2 layer cells
CTRL DEX
**
J
J1092
J1092>bin2-1
J1092>bzr1-D
K
J1092
J1092>bin2-1
J1092>bzr1-D
cells per layer
L1 L2 L3
ns * ** ns
L
meristem size
J1092
J1092>bin2-1
J1092>bzr1-D
ns ***

 © The Author(s)

Figure EV3. Expression patterns of BZR1-family transcription factors in the root and conditional ectopic expression of *bzr1-D* and *bin2-1* in L2 cells.

(**A**) Single-cell based expression patterns of all BZR1-family transcription factors from (https://rootcellatlas.com). Expression patterns are all retrieved with the maximum threshold set at 1.7 (corresponding to the maximum value in *BES1*) for comparability, indicated by the colour scale. Note, *BEH2* expression has been reported to be induced by protoplasting (Denyer et al, 2019). (**B**) Z-stack projection of representative image of the root tip of *pBZR1::BZR1-YFP* plants at 6 dpg. (**C**) Z-stack projection of representative image of the root tip of *pBEH3::gBEH3-eYFP* plants at 6 dpg. (**D**) Z-stack projection of representative image of the root tip of *pBEH4::gBEH4-eYFP* plants at 6 dpg. (**E**) Diagram of the Arabidopsis wild-type root tip, highlighting the *pFEZ::GVG > UAS:mCit-NLS* activated cells upon DEX treatment in blue. (**F**) Z-stack projection of representative images of the root cap of *pFEZ::GVG>bzr1-D* mock treated plants (upper panel) and DEX treated plants (lower panel) at 6 dpg. (**G**) Box plots showing the quantification of the L2 layer cells *pFEZ::GVG>bzr1-D* mock treated (CTRL) plants ($n = 24$) and DEX treated (DEX) plants ($n = 26$) at 6 dpg. Pairwise comparison of the L2 layer cells between CTRL and DEX was analysed using the nonparametric two-tailed Mann–Whitney *U* test (exact *P* value): CTRL vs. DEX, ***$P < 0.001$. (**H**) Z-stack projection of representative images of the root cap of *pFEZ::GVG>bin2-1* mock treated plants (upper panel) and DEX treated plants (lower panel) at 6 dpg. (**I**) Box plots showing the quantification of the L2 layer cells *pFEZ::GVG>bin2-1* mock treated (CTRL) plants ($n = 24$) and DEX treated (DEX) plants ($n = 33$) at 6 dpg. Pairwise comparison of the L2 layer cells between CTRL and DEX was analysed using the nonparametric two-tailed Mann–Whitney *U* test (exact *P* value): CTRL vs. DEX, **$P = 0.001$. (**J**) Representative confocal images of PI-stained root tips of *J1092*, *J1092>bzr1-D and J1092>bin2-1* plants at 6 dpg. Arrowheads indicate first and the last meristematic cell in the cortex file. PI, purple; erGFP, green. (**K**) Box plots showing the quantification of the LRC layer cells (L1, L2 and L3) in *J1092* ($n = 12$), *J1092>bzr1-D* ($n = 12$) and *J1092>bin2-1* ($n = 16$). Statistical differences between the number of cells in each layer were calculated with the nonparametric Kruskal–Wallis test, followed by Dunn's multiple-comparisons post hoc test for pairwise comparisons between the three lines (adjusted *P* values): L1 *J1092* vs. *J1092>bin2-1*, ns ($P = 0.30$); L1 *J1092* vs. *J1092>bzr1-D*, ns$P > 0.99$; L2 *J1092* vs. *J1092>bin2-1*, **$P = 0.010$; L2 *J1092* vs. *J1092>bzr1-D*, *$P = 0.01$; L3 *J1092* vs. *J1092>bin2-1*, **$P = 0.001$; and L3 *J1092* vs. *J1092>bzr1-D*, ns$P > 0.99$. (**L**) Box plots showing the quantification of the meristem size (number of meristematic cortex cells) in J1092 ($n = 8$), *J1092>bzr1-D* ($n = 17$) and *J1092>bin2-1* ($n = 12$). Statistical differences between the number of meristem cells were calculated with the nonparametric Kruskal–Wallis test, followed by Dunn's multiple-comparisons post hoc test for pairwise comparisons (adjusted *P* values): *J1092* vs. *J1092>bin2-1*, ***$P < 0.001$; and *J1092* vs. *J1092>bzr1-D*, ns$P = 0.67$. Scale bars = 50 µm. Box plots show the median (centre line), with the box spanning the first to third quartiles (Q1–Q3); whiskers indicate the minimum (Q0) and maximum (Q4) values. Source data are available online for this figure.

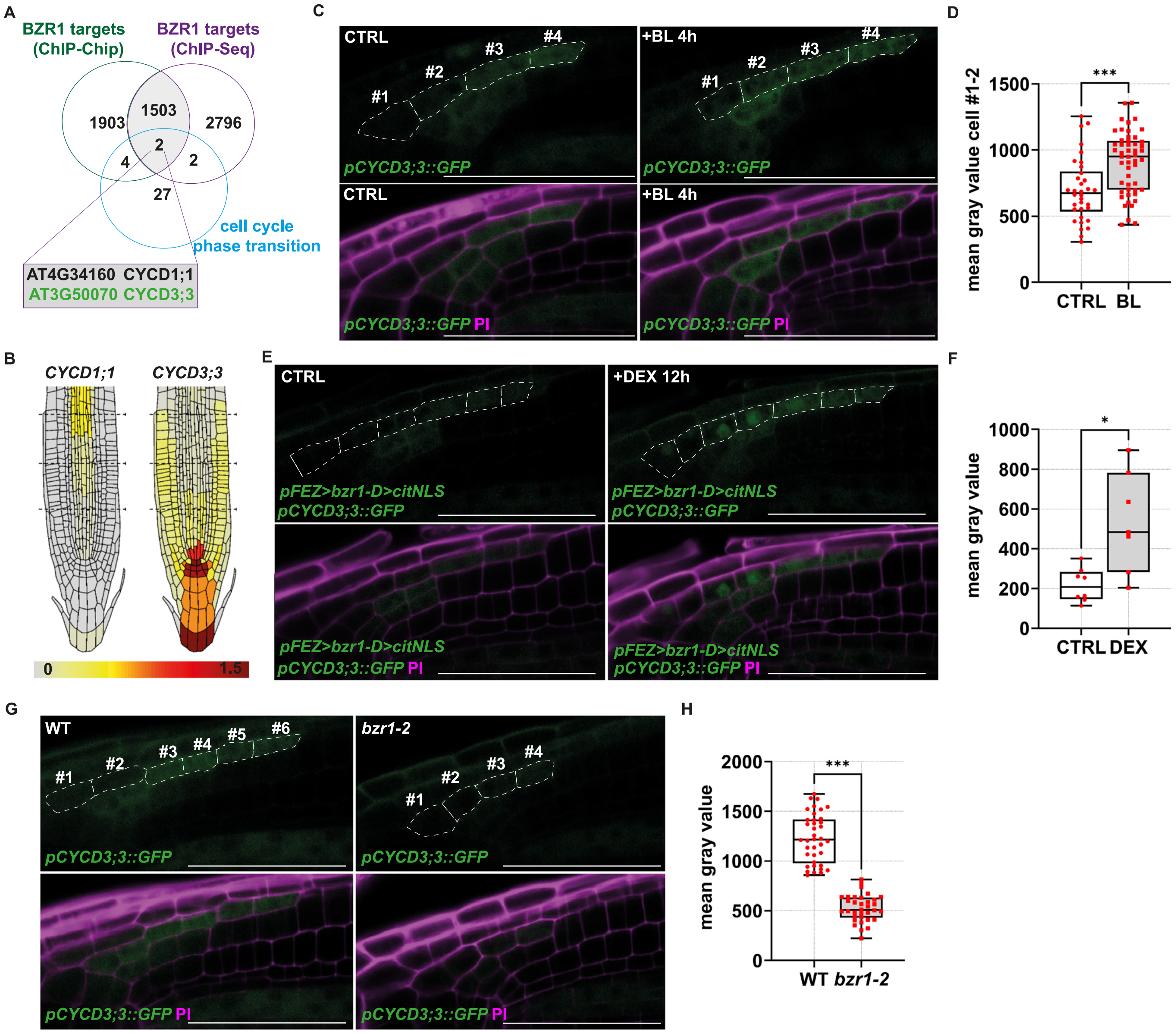

Figure EV4. BR activates *CYCD3;3* expression in L2 cells via BZR1.

(**A**) Venn diagram showing the overlap between high-confidence BZR1 target genes from a ChIP-chip experiment (data ref: Sun et al, 2010; Sun et al, 2010) and a ChIP-Seq experiment (data ref: Oh et al, 2014; Oh et al, 2014) and the gene ontology mitotic cell cycle regulation. Intersecting genes are shown in the table with the corresponding gene identifiers and naming. (**B**) Single-cell expression patterns of *CYCD1;1* and *CYCD3;3* genes in the root meristem tissues from (https://rootcellatlas.org). Expression patterns are all retrieved with the maximum threshold set at 1.5 (corresponding to the maximum value found for *CYCD3;3*) for comparability, as indicated in the colour scale. Note: only *CYCD3;3* mRNA was found expressed in the LRC tissue. (**C**) Representative confocal image of the root cap in *pCYCD3;3::GFP* wild-type plants at 6 dpg at control conditions (CTRL) or after a 4-h treatment with 100 nM epibrassinolide (+BL). In the upper GFP channel, the L2 cells are marked and numbered starting from the most distal cells. Note that BL activates *GFP* expression mainly in cells #1 and #2 of the L2 layer. (**D**) Box plots showing the mean grey value quantification of GFP intensity (cell #1 and #2 of the L2 layer) in *pCYCD3;3::GFP* CTRL ($n = 34$) and +BL ($n = 51$). Statistical differences between CTRL and +BL were assessed using Welch's *t* test (*P* value): CTRL vs. +BL, $***P < 0.001$. (**E**) Representative confocal image of the root cap from the F1 progeny of a cross between *pFEZ>bzr1-D>citNLS x pCYCD3;3::GFP* at 6 dpg under control condition (CTRL) or after 12-h induction with dexamethasone (+DEX). PI-staining magenta, and GFP signal is shown in green. (**F**) Box plots showing quantification of the mean grey value of GFP intensity (only cell #1 and #2 of the L2 layer) in *pFEZ>bzr1-D>citNLS x pCYCD3;3::GFP* CTRL ($n = 8$) and +DEX ($n = 7$). Statistical differences between CTRL and +BL were assessed using Welch's *t* test (*P* value): CTRL vs. +DEX, $*P = 0.01$. (**G**) Representative confocal image of the root cap from *pCYCD3;3::GFP* wild-type and in the *bzr1-2* mutant background at 6 dpg. PI-staining magenta, and GFP signal is shown in green. In the upper GFP channel, the L2 cells are marked and numbered starting from the most distal cells. (**H**) Box plots showing the quantification of the mean grey value of GFP intensity (whole L2 layer) in *pCYCD3;3::GFP* WT ($n = 38$) and *pCYCD3;3::GFP bzr1-2* plants ($n = 34$). Statistical differences between CTRL and +BL were assessed using Welch's *t* test (*P* value): WT vs. *bzr1-2*, $***P < 0.001$. Scale bars = 50 μm. Box plots show the median (centre line), with the box spanning the first to third quartiles (Q1–Q3); whiskers indicate the minimum (Q0) and maximum (Q4) values. Source data are available online for this figure.

© The Author(s)
